# Genomic erosion in a demographically recovered bird species during conservation rescue

**DOI:** 10.1111/cobi.13918

**Published:** 2022-05-12

**Authors:** Hazel A. Jackson, Lawrence Percival‐Alwyn, Camilla Ryan, Mohammed F. Albeshr, Luca Venturi, Hernán E. Morales, Thomas C. Mathers, Jonathan Cocker, Samuel A. Speak, Gonzalo G. Accinelli, Tom Barker, Darren Heavens, Faye Willman, Deborah Dawson, Lauren Ward, Vikash Tatayah, Nicholas Zuël, Richard Young, Lianne Concannon, Harriet Whitford, Bernardo Clavijo, Nancy Bunbury, Kevin M. Tyler, Kevin Ruhomaun, Molly K. Grace, Michael W. Bruford, Carl G. Jones, Simon Tollington, Diana J. Bell, Jim J. Groombridge, Matt Clark, Cock Van Oosterhout

**Affiliations:** ^1^ Durrell Institute of Conservation and Ecology, School of Anthropology and Conservation University of Kent Canterbury UK; ^2^ NIAB Cambridge UK; ^3^ School of Environmental Sciences University of East Anglia Norwich UK; ^4^ The Earlham Institute Norwich UK; ^5^ School of Biological Sciences University of East Anglia Norwich UK; ^6^ Department of Zoology, Faculty of Science King Saud University Riyadh Saudi Arabia; ^7^ Department of Life Sciences The Natural History Museum London UK; ^8^ GLOBE Institute University of Copenhagen Copenhagen Denmark; ^9^ Department of Crop Genetics John Innes Centre Norwich UK; ^10^ Institute of Zoology Zoological Society of London London UK; ^11^ NERC Biomolecular Analysis Facility, Department of Animal and Plant Sciences University of Sheffield Sheffield UK; ^12^ Mauritian Wildlife Foundation Vacoas‐Phoenix Mauritius; ^13^ Durrell Wildlife Conservation Trust Jersey Channel Islands; ^14^ Seychelles Islands Foundation Victoria Seychelles; ^15^ Centre for Ecology and Conservation University of Exeter Penryn UK; ^16^ Norwich Medical School University of East Anglia Norwich UK; ^17^ National Parks and Conservation Service, Ministry of Environment Government of Mauritius Réduit Mauritius; ^18^ Molly K. Grace, Department of Zoology University of Oxford Oxford UK; ^19^ School of Biosciences Cardiff University Cardiff UK; ^20^ North of England Zoological Society Chester Zoo Chester UK

**Keywords:** captive breeding, genetic diversity, genetic management, genetic rescue, *Nesoenas mayeri*, diversidad genética, manejo genético, reproducción en cautiverio, rescate genético, *Nesoenas mayeri*

## Abstract

The pink pigeon (*Nesoenas mayeri*) is an endemic species of Mauritius that has made a remarkable recovery after a severe population bottleneck in the 1970s to early 1990s. Prior to this bottleneck, an ex situ population was established from which captive‐bred individuals were released into free‐living subpopulations to increase population size and genetic variation. This conservation rescue led to rapid population recovery to 400–480 individuals, and the species was twice downlisted on the International Union for the Conservation of Nature (IUCN) Red List. We analyzed the impacts of the bottleneck and genetic rescue on neutral genetic variation during and after population recovery (1993–2008) with restriction site‐associated sequencing, microsatellite analyses, and quantitative genetic analysis of studbook data of 1112 birds from zoos in Europe and the United States. We used computer simulations to study the predicted changes in genetic variation and population viability from the past into the future. Genetic variation declined rapidly, despite the population rebound, and the effective population size was approximately an order of magnitude smaller than census size. The species carried a high genetic load of circa 15 lethal equivalents for longevity. Our computer simulations predicted continued inbreeding will likely result in increased expression of deleterious mutations (i.e., a high realized load) and severe inbreeding depression. Without continued conservation actions, it is likely that the pink pigeon will go extinct in the wild within 100 years. Conservation rescue of the pink pigeon has been instrumental in the recovery of the free‐living population. However, further genetic rescue with captive‐bred birds from zoos is required to recover lost variation, reduce expression of harmful deleterious variation, and prevent extinction. The use of genomics and modeling data can inform IUCN assessments of the viability and extinction risk of species, and it helps in assessments of the conservation dependency of populations.

## Resumen

La paloma rosada (*Nesoenas mayeri*) es una especie endémica de Mauricio que se ha recuperado impresionantemente después de un grave cuello de botella poblacional a principios de la década de 1970 que duró hasta inicios de la década de 1990. Antes de este cuello de botella se había establecido una población ex situ de la cual se liberaban individuos reproducidos en cautiverio a las subpoblaciones en libertad para incrementar la variación genética y el tamaño poblacional. Este rescate de conservación derivó en una recuperación rápida de la población (400‐480 individuos) y la especie cambió positivamente de categoría dos veces en la Lista Roja de la Unión Internacional para la Conservación de la Naturaleza (UICN). Analizamos los impactos del cuello de botella y el rescate genético sobre la variación genética neutral durante y después de la recuperación poblacional (de 1993 a 2008) mediante secuenciación RAD, análisis de microsatélites y análisis genéticos cuantitativos de los datos del libro genealógico de 1112 aves ubicadas en zoológicos de Europa y los Estados Unidos. Usamos simulaciones por computadora para estudiar los cambios pronosticados en la variación genética y en la viabilidad poblacional del pasado hacia el futuro. La variación genética declinó rápidamente, a pesar de la recuperación poblacional, y el tamaño efectivo de la población fue aproximadamente un orden de magnitud más pequeño que el tamaño del censo. La especie contó con una carga genética elevada de casi 15 equivalentes letales para la longevidad. Nuestras simulaciones pronostican que la endogamia continua probablemente resultará en un incremento en la expresión de mutaciones deletéreas (es decir, una carga realizada elevada) y en una depresión endogámica severa. Sin acciones continuas para la conservación, es probable que la paloma rosada esté extinta en vida libre dentro de cien años. El rescate de conservación de la paloma rosada ha sido fundamental en la recuperación de la población silvestre; sin embargo, se requiere de un rescate genético adicional con las aves de reproducción en cautiverio de los zoológicos para recuperar la variación perdida, reducir la expresión de la variación deletérea dañina y prevenir la extinción. El uso de la genómica y los datos modelados puede orientar las valoraciones de la UICN sobre la viabilidad y el riesgo de extinción de las especies, además de que ayuda en la evaluación de la dependencia que tienen las poblaciones de la conservación.

## INTRODUCTION

**FIGURE 1:**
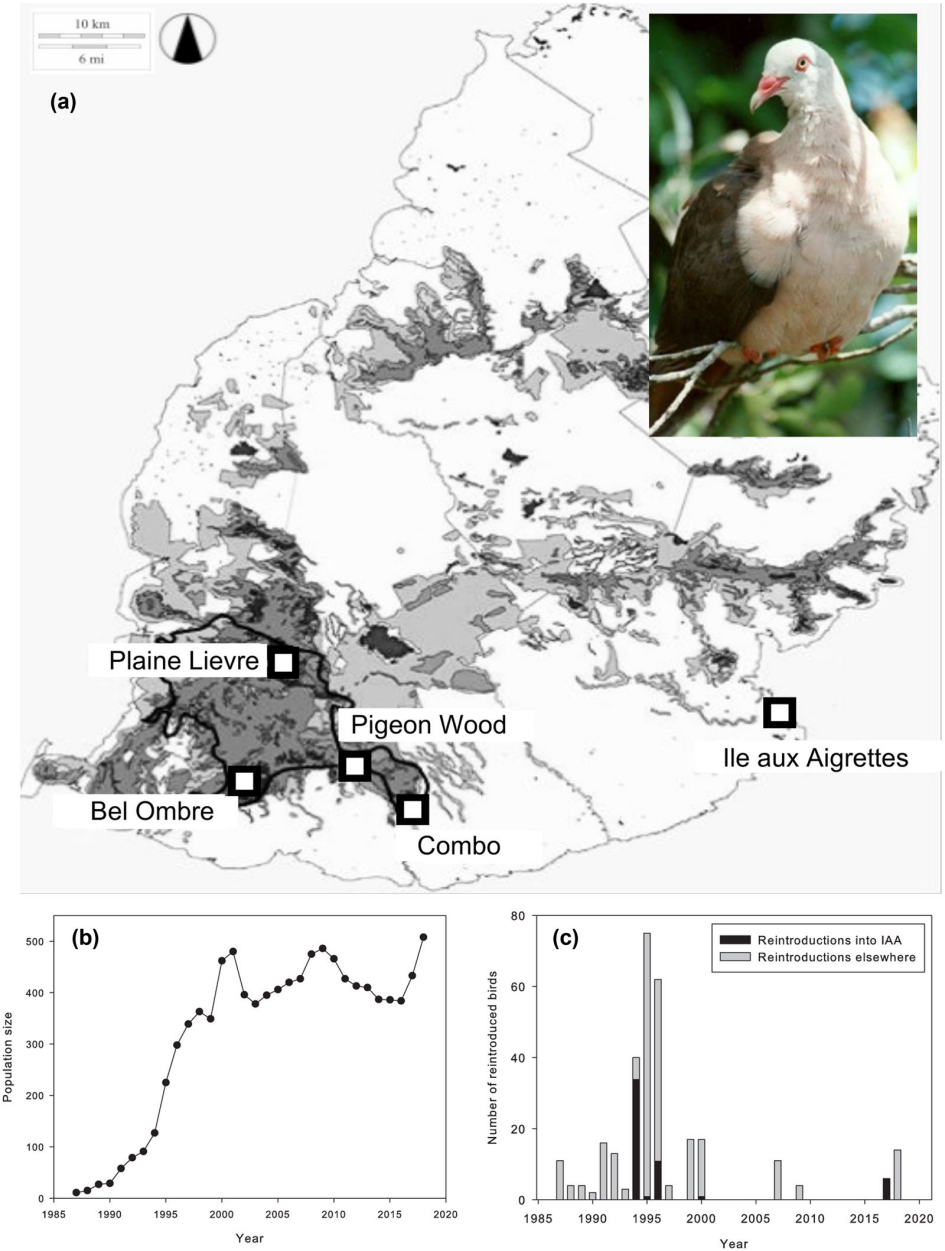
(a) Location of 5 subpopulations of pink pigeon (pictured) on Mauritius in 2008 (squares) (black polygon, Black River Gorges National Park; shading, forest; inset, adult bird), (b) population size, derived from field monitoring, of the free‐living Mauritius pink pigeon population over time (bottleneck and recovery), and (c) number of captive‐bred pink pigeons released in the Ile aux Aigrettes (IAA) population and in other free‐living populations during the species recovery program

The pink pigeon is an endemic species of Mauritius that experienced a population decline over several centuries due to habitat fragmentation and destruction and invasive species (Jones, 2013; Jones & Swinnerton, 1997). These factors reduced the free‐living population to approximately 10 individuals by 1990 (Jones, 2013) (Figure 1). From 1976 to 1981, 12 individuals were taken from the last free‐living population at Pigeon Wood to establish a captive breeding population at the Gerald Durrell Endemic Wildlife Sanctuary (GDEWS), Mauritius. The GDEWS gene pool has been intensively managed, with genetic diversity conserved through careful captive breeding and genetic supplementation with birds from the free‐living subpopulations and the captive population in Jersey Zoo (United Kingdom) (Jones, 2013; Swinnerton et al., 2004).

The GDEWS has been a source of genetic variation for the free‐living metapopulation, and it was used to establish all current subpopulations, except for the subpopulation in Pigeon Wood, which survived in the wild in the 1970s (Swinnerton et al., 2004). The pink pigeon conservation program thus comprised reintroduction, demographic rescue, and genetic supplementation. After 4 decades of intensive management, the free‐living metapopulation reached a near‐stable size of around 400 individuals in 2013 (Jones, 2013) (Figure 1), resulting in its downlisting twice on the International Union for the Conservation of Nature (IUCN) Red List from critically endangered to vulnerable (IUCN, 2018). However, the genomic consequences of the bottleneck and conservation program have not been fully evaluated (but see Swinnerton et al., 2004; Albeshr, 2016; Ryan, 2020).

We studied the pink pigeon to evaluate the conservation impact and long‐term population viability of this threatened species via RAD‐sequencing and microsatellite genotyping data. We also estimated the genetic load of the captive population and used these data in several modeling approaches to forecast population viability and extinction risk under different conservation management scenarios. We considered the benefits of complementing the IUCN Red List assessment with assessment of the IUCN Green Status of Species (i.e., Grace et al., 2021) (IUCN, 2021) by including genomics and modeling data, arguing that this would improve the evaluation of the conservation impact and management dependency of recovered species.

## METHODS

### Genomic analyses

Genome‐wide genetic diversity of the free‐living pink pigeon metapopulation was analyzed by restriction site‐associated DNA sequencing (RAD‐seq) of 175 birds from 5 subpopulations: Ile aux Aigrettes (IAA) (*n* = 116), Plaine Lievre (PL) (*n* = 12), Bel Ombre (BO) (*n* = 12), Pigeon Wood (PW) (*n* = 10), and Combo (CO) (*n* = 25) sampled from 1994 to 2008.

The DNA from an individual that died naturally was used to construct PCR‐free paired end libraries suitable for DISCOVAR de novo contig assembly (Weisenfeld et al., 2014). Size‐selected Illumina Nextera Mate Pair library (Heavens et al., 2015) reads were classified with NextClip (Leggett et al., 2014) and the DISCOVAR contigs were scaffolded with SOAPdenovo (Li et al., 2010). The resulting assembly had an N50 of 8 Mbp and was 94.38% complete based on analyses with BUSCO 3.1.0 (Waterhouse et al., 2018) of 4915 avian genes (aves_odb9 database) (details in Appendix S1).

Ethanol‐preserved blood was resuspended in Tris‐EDTA (TE) buffer solution, prior to DNA extraction, using the Agencourt GenFind V2 Blood & Serum Genomic DNA Isolation Kit. The RAD‐seq libraries (SbfI digests) were constructed from each DNA sample (Hohenlohe et al., 2010), and a custom Illumina recipe was used for sequencing (details in Appendix S3).

We modified settings for Illumina's BCL2FASTQ conversion script to account for the custom run metrics, and we used RADplex to demultiplex (Leggett et al., 2013). Reads containing the SbfI overhang were aligned to the pink pigeon genome with BWA‐MEM (Li, 2013); genotypes were called using SAMtools (Li et al., 2009), BCFtools (Li, 2011), and VCFtools (Danecek et al., 2011). Genotype and studbook data were used in PLINK (Purcell et al., 2007) to exclude markers on sex chromosomes. SplitsTree 4 (Huson & Bryant, 2006) generated a NeighbourNet network from 43,967 single nucleotide polymorphisms (SNPs) for relatedness scores (details in Appendix S3).

The pink pigeon genome was superscaffolded into pseudochromosomes by alignment to the reference genome of the zebra finch (*Taeniopygia guttata*) with RaGOO (1.1) (Alonge et al., 2019). We used VCFtools to convert genotype coordinates and BCFtools to calculate runs of homozygosity (ROH). The ROH intervals were calculated in R, and heterozygosity was plotted per chromosome after the method of Kardos et al. (2018) (details in Appendix S1).

### Pedigree analyses

A pedigree file (.PED, http://zzz.bwh.harvard.edu/plink/data.shtml#ped) recapitulating the families in the IAA population was created in a Jupyter notebook with the pandas framework (Mckinney, 2011) and the graph‐oriented networkx Python library (Hagberg et al., 2008). Briefly, the networkx library was used to create a directional graph of all known individuals, which was then traversed to obtain the 12 different known families.

### Microsatellite DNA analyses

A total of 659 birds, sampled from 1993 to 1997 (hereafter 1990s) and 2003 to 2011 (2000s), were genotyped at 22 loci. The samples comprised 571 free‐living birds from 5 subpopulations in Mauritius, 36 birds from the captive GDEWS in Mauritius, 25 birds from U.S. zoo collections, and 27 birds from European zoos (Appendix S2). Mean unbiased expected heterozygosity (*H*_e_) was calculated in GenAlEx 6.5 (Peakall & Smouse, 2006) and allelic richness (*A*_r_) was calculated using rarefaction. Effective population size (*N*_e_) was calculated for the entire free‐living metapopulation from the earliest year of sampling (1993) to the latest year (2010) and for each subpopulation for the 2 sample groups (1990s and 2000s) with the linkage disequilibrium method (LDM) in NeEstimator 2.01 (Do et al., 2014). A minor allele threshold of *P*_crit_ = 0.02 was applied to reduce bias (Waples & Do, 2008), and 95% confidence intervals were calculated using the parametric option (Waples, 2006).

Genetic differentiation, *F*_ST_ and *D*_JOST_, between captive populations and subpopulations of pink pigeons of the 1990s and 2000s was calculated with GenAlEx (Peakall & Smouse, 2006). A Bayesian clustering approach, in STRUCTURE 2.3.4 (Pritchard et al., 2000), was used to detect the most likely number of genetic clusters (*K*) among captive and wild subpopulations in the 1990s and 2000s. The most likely number of clusters were identified by evaluating log likelihood and delta *K* scores (Evanno et al., 2005) and by calculating estimators based on a count of the number of independent clusters from user‐defined groups (Puechmaille, 2016) in Structure Selector (Li & Liu, 2018). Gene flow between subpopulations was calculated using a Bayesian approach in BayesAss (Wilson & Rannala, 2003).

We employed additional computer simulations coded in a Minitab 12.1 macro to evaluate the effects of genetic supplementation on microsatellite variation. Using 52 captive birds from Europe and the United States genotyped at 22 loci, we examined the impact of reintroducing a random subsample of those birds (*n* = 5, 10, …, 50) on the genetic variation in the combined gene pool of 64 free‐living pink pigeons sampled in 2010. We quantified the effects of reintroduction by analyzing the effective number of alleles (*A*_e_) and the actual number of alleles (*A*).

### Genetic load

The genetic load was calculated for adult birds that died from 1976 to 21 December 2018: 1112 birds out of 1308 birds in the studbook. The data were filtered in PMX's Genetic Module to include only captive‐born birds with known inbreeding coefficients and known age at death. The genetic load was expressed as the number of lethal equivalents (LEs) and calculated using a logistic regression of (ln transformed) longevity of birds (number of days lived at death + 1) against inbreeding coefficient (*F*). The inbreeding coefficient was calculated based on studbook data with PMX 1.4.2 (Ballou et al., 2011). The number of LEs was calculated using ln(longevity) = *A* – *BF*, where *A* is the intercept on the *y*‐axis (i.e., the ln‐transformed longevity of noninbred birds) and *B*, the slope of the regression line, equals the number of LEs in a haploid gamete. In a diploid individual, 2*B* equals the number of LEs (Frankham, 2005).

### Vortex simulations to assess extinction risk

To assess the mid‐ to long‐term population viability of the free‐living pink pigeon metapopulation, we used Vortex 10.1 (Lacy & Pollak, 2014) to simulate 3 management scenarios. Scenario 1 represented the free‐living metapopulation without supplementation. Scenario 2 (demographic rescue) represented introduction of birds from a hypothetical captive population with the same allele frequencies as the free‐living metapopulation in Mauritius. Scenario 3 (genetic rescue) simulated the genetic supplementation of the metapopulation with individuals from the captive population containing novel alleles and a low mean kinship with the free‐living metapopulation. We had no population genomic data on the captive source population with which to calculate kinship between captive and wild birds, and the relatedness between these gene pools may by higher than we assumed, which would reduce the impact of genetic rescue. Furthermore, our population genomic and quantitative genetic analyses were conducted using samples collected over 10 years ago, and the gene pool will have changed since then. Consequently, scenario 3 (genetic rescue) simulated a best‐case scenario, and the actual impact of genetic rescue may be less than predicted here.

Each scenario was run for 100 years and averaged across 1000 iterations. In the Vortex simulations, we assigned an inbreeding coefficient of *F* = 0.15 for the free‐living metapopulation (Swinnerton et al., 2004). Inbreeding depression was modeled for all scenarios based on the demography and biology of the pink pigeon (Appendix S3a). The model was parameterized with empirical data gathered from the long‐term (∼40 years) study of the pink pigeon. The following variables were most challenging to parameterize: carrying capacity (uncertainty), juvenile mortality (data were from a single subpopulation and applied to all subpopulations), number of LEs (estimated based on longevity data in captivity), and percentage of females with 1 offspring (data from captive birds applied to the wild population). To ensure that the final model was robust, accurate, and useful, we explored the parameter space of these variables (Pe'er et al., 2013; Pacioni et al., 2017) with single‐factor sensitivity tests in Vortex. We used scenario 1 as the baseline model, testing the impact of uncertainty in these parameters (Appendices S3l and S3m). Each sensitivity‐test scenario was run for 1000 iterations over 100 years. To test for statistical significance between scenarios and evaluate the results of the sensitivity testing, the strictly standardized mean difference was calculated in VortexR 1.1.5 (Pacioni & Mayer, 2017) in R 3.5.0 (R Core Team, 2018).

### SLiM simulations to assess conservation dependency

We performed individual‐based forward simulations with SLiM 3.1 (Haller & Messer, 2019) to examine the impact of different management regimes. We modeled neutral genetic variation and a genetic load of ∼15 LEs, which simulated our empirical data. Details about the parameterization of the model are in Appendix S4. Briefly, we simulated an ancestral population of 16,000 individuals (Ryan, 2020) that had a slow population collapse followed by a severe recent bottleneck (Appendix S4). The simulated demographic trajectory was informed by the inferred (pre‐1980s) population size and recorded census trajectories from 1980 to 2020 (Appendix S4). The trajectories were maintained for another 100 years (to 2120) to simulate future dynamics. We modeled 1 wild population and 1 captive population founded by 12 individuals in 1976 that grew at the rate reported in GDEWS records until reaching an average of 120 individuals. Reproductive age, fecundity, and mortality were drawn from a distribution that reflected the productivity of pink pigeons in the wild and captivity (Appendix S4). This resulted in an average generation time of 3.5 simulation steps, similar to the generation time of the pink pigeon.

We tested 4 scenarios after the population reached the bottom of the bottleneck: (1) no intervention, wild population remained at the bottleneck size; (2) demographic rescue, population increased to 400 individuals as recorded in the wild population, but without contribution from the captive population; (3) genetic rescue, wild population received translocations from the captive population but remained at a low size; and (4) genetic and demographic rescue, which was most similar to the conservation rescue of the pink pigeon. Translocations occurred at rates reported by GDEWS until 2019 (Appendix S4a) and thereafter at random to capture the same dynamics (Appendix S4a). We recorded temporal changes in neutral nucleotide diversity and the realized load (i.e., the expressed deleterious mutations), expressed in LEs (Bertorelle et al., 2022). To incorporate the effects of extinction, replicates that went extinct before 2120 contributed zero values (*π* = 0) to nucleotide diversity (neutral variation) in all years after population extinction. Extinct populations contributed a realized load identical to that recorded at the time of their extinction, and this load was used for all subsequent years. Due to the extremely high computational demands, we simulated only 40 replicates and report the 90% confidence limits of the summary statistics. Details on the SLiM model are in Appendix S4.

### Code availability

Code is availabe from https://github.com/pink‐pigeon‐conservation/Genomic_erosion_during_conservation_rescue.

## RESULTS

### Genetic drift

**FIGURE 2:**
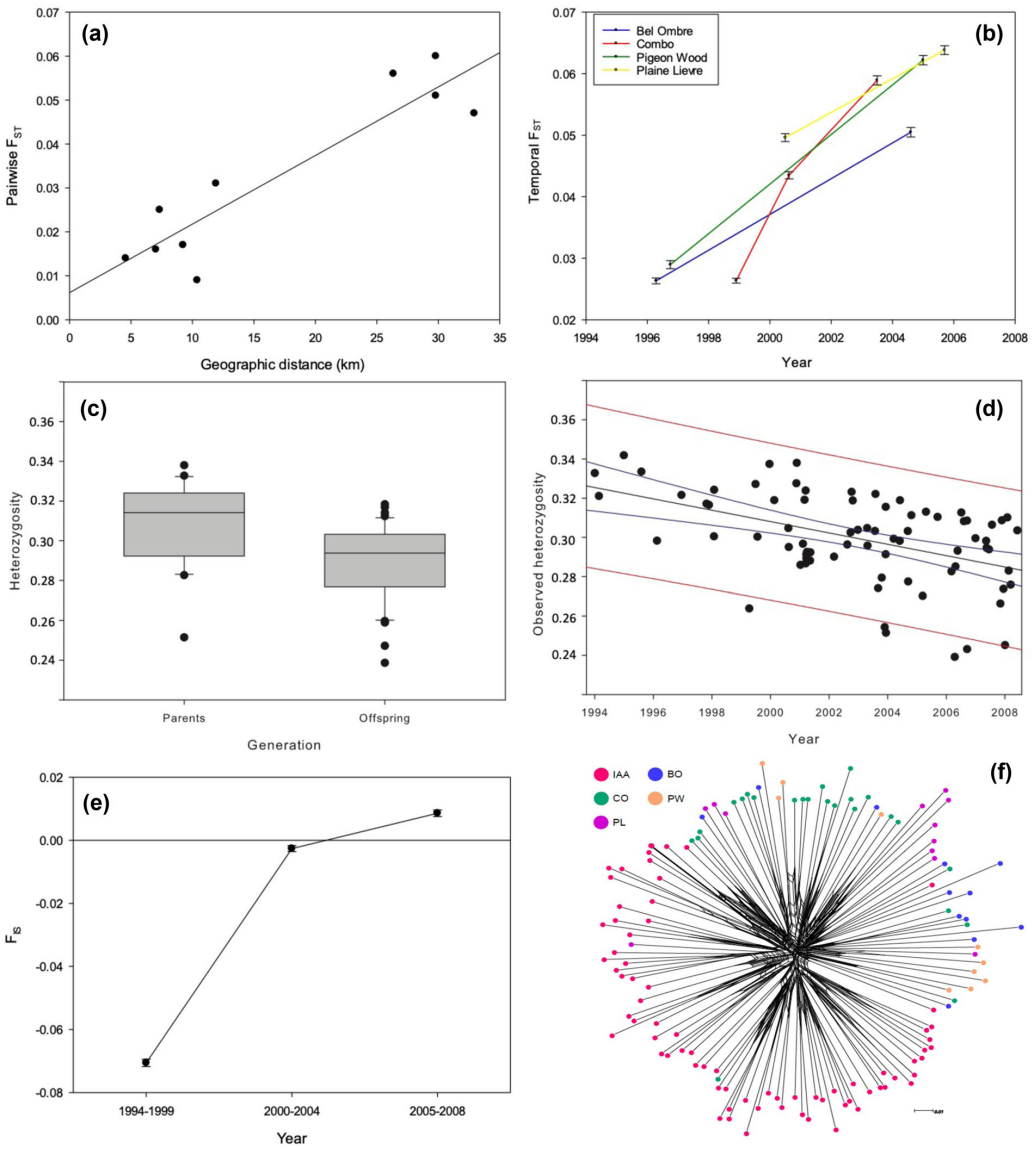
Relationship between genetic divergence (pairwise *F*_ST_) and (a) geographic distance between populations (regression: *F*_1,8_ = 42.14, *p* < 0.001, *R* ^2^ = 84.0%) and (b) genetic divergence (mean and SE) between the Ile aux Aigrettes (IAA) population and Bel Ombre, Combo, Pigeon Wood, and Plaine Lievre populations of pink pigeon over time; (c) observed heterozygosity (*H*_o_) between the parent (*H*_o_ = 0.314) and offspring (*H*_o_ = 0.294) generations over time, based on RAD‐seq data, in the IAA population of pink pigeons; (d) heterozygosity in birds hatched from 1994 to 2008, based on RAD‐seq data, in the IAA population (regression: *F*_1,73_ = 21.41, *p* < 0.001, *R* ^2^ = 22.7%); (e) temporal change in inbreeding coefficient (*F*_IS_) (mean and SE) (i.e., observed heterozygosity) for pink pigeons from the (IAA) population; and (f) neighbor‐net network of 133 pink pigeon samples (tips colored by population)

Population genetic analysis of 43,967 loci revealed considerable geographic structure consistent with isolation by distance (regression: *F*_1,8_ = 42.14, *p* < 0.001, *R* ^2^ adjusted = 82.1%) (Figure 2a). However, this isolation‐by‐distance signal was caused by the IAA subpopulation on an isolated island. The IAA was the most distant subpopulation in our data set (Figure 1a) and the most genetically diverged. When excluding the IAA, the isolation‐by‐distance signal disappeared (regression: *F*_1,4_ = 0.59, *p* = 0.486).

The genetic differentiation between subpopulations increased over time, consistent with strong genetic drift (Figure 2b). Genetic drift and inbreeding also caused a rapid loss of genome‐wide heterozygosity in the IAA subpopulation in 2005−2008 (Appendix S3f); the observed heterozygosity (*H*_o_) of 44 offspring (0.294) was significantly lower than that of the 20 parental birds (*H*_o_ = 0.314) (Kruskal–Wallis test: *H* = 10.72; df = 1; *p* = 0.001) (Figure 2c). The 6.15% loss of heterozygosity in a single generation is expected for a population with *N*_e_ = 7.6, which compares to a census subpopulation size (*N*_c_) of 70–90 in those years and an *N*_e_/*N*_c_ = 0.084–0.109. Furthermore, the observed genome‐wide heterozygosity declined steeply from 1994 to 2008 in the IAA subpopulation (Figure 2d) and in other subpopulations (Appendices S3f and S3g).

### Demographic rescue

From 1994 to 1996, 47 birds from GDEWS were translocated to form the IAA subpopulation (Figure 1c). Both RAD‐seq data and pedigree data were available for 109 IAA birds, and we detected a significant relationship between the pedigree‐calculated inbreeding coefficient and the genome‐wide heterozygosity (regression: *F*_1,108_ = 45.02, *p* < 0.0001, adjusted *R* ^2^ = 29.0%) (Appendix S3g). Individuals that hatched between 1994 and 1999 were significantly less inbred than expected from a randomly mating population, and these birds showed an excess in *H*_o_ (*F*_IS_ = −0.07059 [SE 0.00115]; 1‐sample *t* test: *t* = −61.23, *p* < 0.0001) (Figure 2e). Individuals born from 2000 to 2004 were the offspring produced by random mating in the IAA subpopulation, and this cohort was in Hardy–Weinberg equilibrium. However, birds that hatched from 2005 to 2008 were more inbred and less heterozygous than expected from panmixia (mean *F*_IS_ = 0.00857 [0.00104]; *t* = 8.25, *p* < 0.0001) (Figure 2e). This suggests that soon after supplementation stopped in 1996 (Figure 1c), allele segregation returned to Mendelian proportions (Figure 2e), after which inbreeding commenced (2005−2008). Meanwhile, genetic drift continued to erode variation in the IAA subpopulation.

### Gene flow and population bottleneck

**FIGURE 3:**
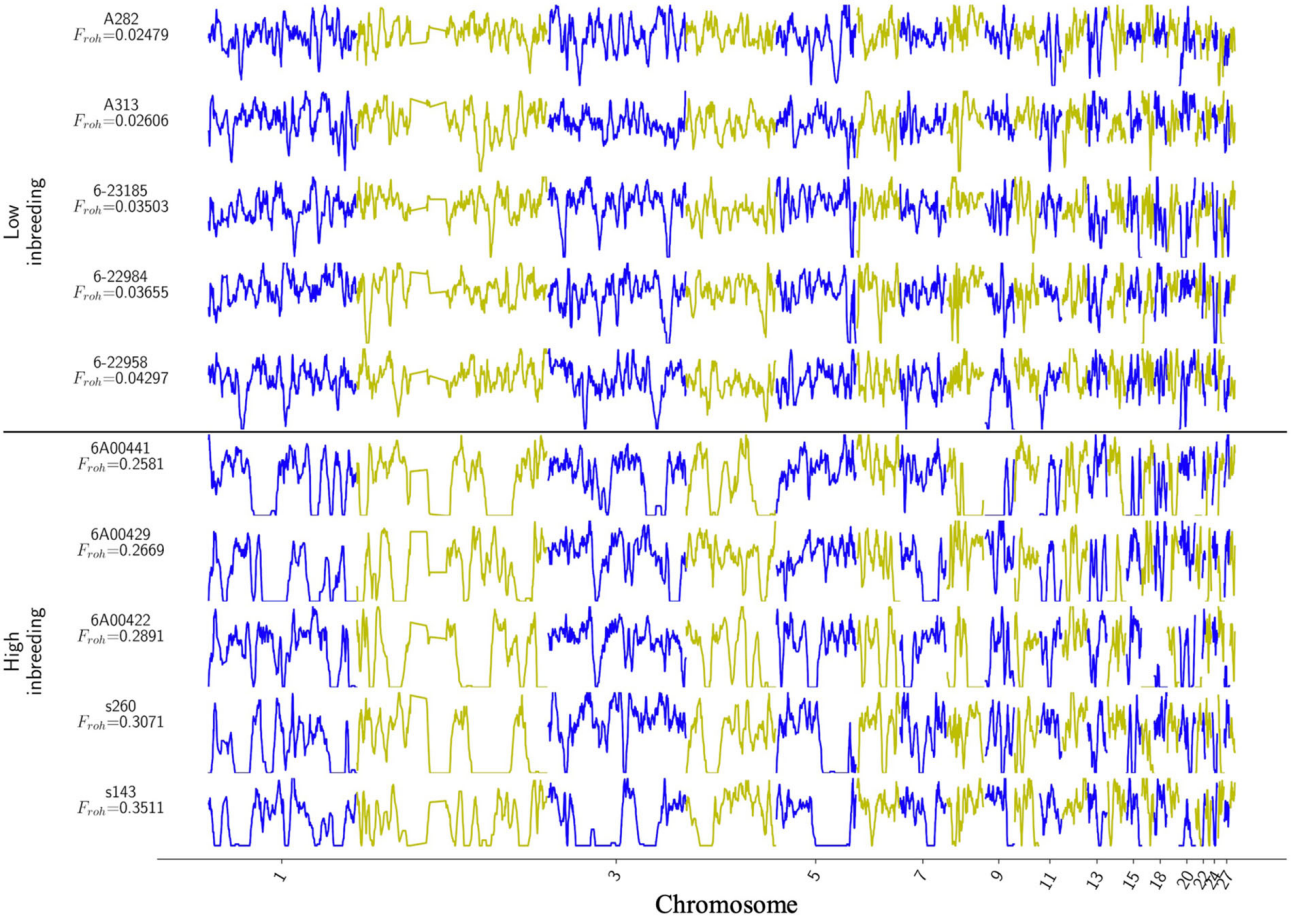
Runs of homozygosity (ROH) in the pink pigeon genome across chromosome scaffolds 1–27. Highly inbred individuals (*F*_ROH_ > 0.25, i.e., a level of inbreeding higher than after a single full‐sib mating) show long ROH, occasionally spanning the length of nearly half a chromosome. Numbers and letters above *F*_ROH_ refer to pink pigeon identification number in the studbook

There was very limited genetic differentiation among subpopulations, as demonstrated by the low pairwise *F*_ST_ values, which ranged from 0.009 (PW vs. BO) to 0.053 (PL vs. IAA). This was further supported by the coancestry analyses (Appendix S3). The population genetic signature of the bottleneck was evident in the star‐like structure of the network (extensive loops at the center) (Figure 2f). The close relationship and genetic similarity of individuals across different subpopulations was illustrated by our DensiTree figure (Appendix S3j), which was characterized by a highly interconnected network with short terminal branches. The impact of inbreeding on genome‐wide variation was perhaps best illustrated by ROH in the pink pigeon genome (Figure 3). Highly inbred birds (*F* > 0.25, i.e., a level of inbreeding higher than that after a single full‐sib mating) had long ROH, occasionally spanning the length of nearly half a chromosome (Figure 3).

### Population genetic analyses with microsatellite markers

Expected heterozygosity and allelic richness declined by 8−16% and 6−12%, respectively, in all free‐living subpopulations from 1995 to 2011 (Appendix S2). The captive zoo populations diverged significantly from the free‐living subpopulations. Not a single free‐living or captive GDEWS bird from Mauritius was assigned to the zoo population clusters (Appendix S2). The estimates of *N*_e_ of each subpopulation dropped to *N*_e_ ≤ 40 by 2010, when the *N*_e_ of the entire free‐living metapopulation was fewer than 50 birds (Appendix S2). These *N*_e_ estimates corresponded well with the total metapopulation size summed across subpopulations estimated using the RAD‐seq data. Migration rates in the 1990s and 2000s were relatively low between subpopulations, which explained why genetic differentiation increased over time (Appendix S2). A simulation model showed that genetic supplementation with individuals from the captive populations in zoos substantially increased the number of microsatellite alleles and effective number of alleles in the free‐living subpopulation in Mauritius (Appendix S2).

### Genetic load and Vortex simulations

**FIGURE 4:**
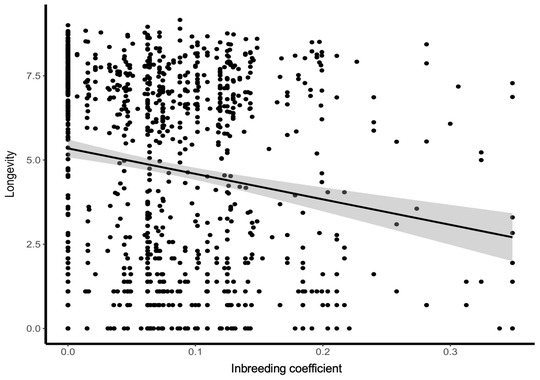
Longevity of a pink pigeon (in days) relative to its inbreeding coefficient (*F*_1, 1111_ = 33.550, *p* < 0.0001). The mean number of lethal equivalents (LEs) equals 15.13 (5–95% CI, 10.00–20.25)

**FIGURE 5:**
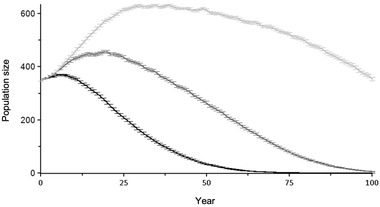
Predicted mean (SE) census population size (*N*) over 1000 iterations of free‐living pink pigeons modeled in Vortex (Lacy & Pollak, 2014) for each year of the model and 3 management scenarios (black line, scenario 1 no supplementation; dark gray line, scenario 2 demographic rescue [i.e., populaiton supplementation with hypothetical gene pool similar to the free‐living metapopulation]; light gray line, scenario 3 genetic rescue [i.e., supplementation with zoo‐bred captive birds]). The supplementation regimes for scenarios 2 and 3 are identical, that is, 10 birds for each subpopulaiton every 5 years

The captive population had a high genetic load equal to a mean number of LEs of 15.13 (5–95% confidence interval [CI], 10.00–20.25) (Figure 4). We parameterized Vortex with 15 LEs and simulated 3 management scenarios. Sensitivity testing showed that the model was relatively robust to fluctuations in parameterization (values <25% different to the baseline scenario), although 3 of the tested 34 scenarios resulted in significant difference in the abundance of birds by year 100 (see Appendix S3n). Scenario 1 (no supplementation) resulted in likely extinction in the wild within the next 50−100 years (Figure 5). Scenario 2 (demographic rescue) reduced extinction risk (Figure 5); but still, extinction was projected to occur within the next ∼100 years. Under scenario 3 (demographic and genetic rescue), inbreeding depression was alleviated and the number of birds increased, thereby reducing extinction probability. Although scenario 2 (demographic rescue) also appeared to improve the outcome for the pink pigeon population (Figure 5), scenario 3 was the only model that produced a result that was significantly different from the base scenario of no supplementation (Appendix S3e).

**FIGURE 6:**
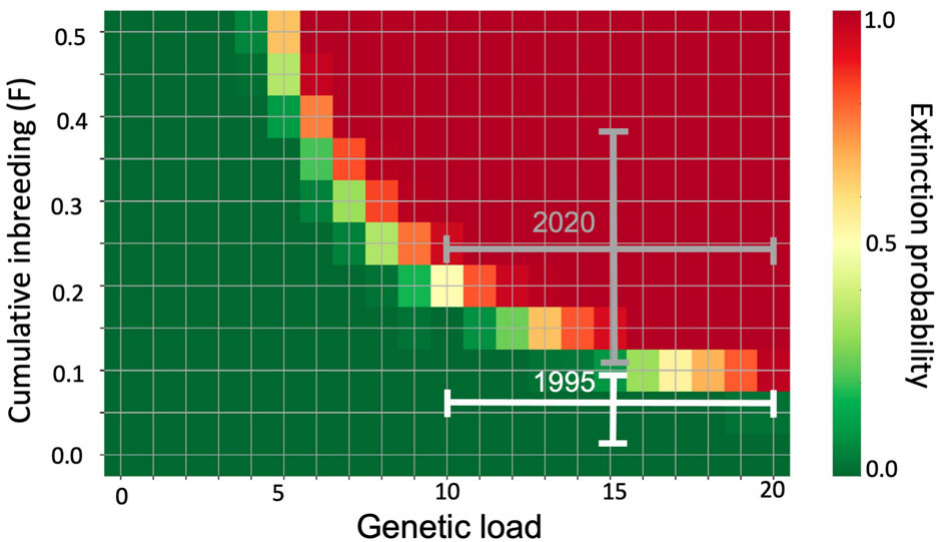
Relationship between the genetic load (expressed in lethal equivalents [LEs]), inbreeding coefficient (*F*), and the probability of extinction after 100 years for the free‐living population of pink pigeon modeled in Vortex (Lacy & Pollak, 2014). The population increased in mean (5–95% CI) inbreeding coefficient between 1995 (white bars) and 2020 (gray bars), which also increased its extinction probability

Varying the level of inbreeding and genetic load on the 100‐year extinction probability resulted in the population likely becoming extinct within the next 100 years (Figure 6). In Figure 6, the level of inbreeding in 1995 was calculated using the IAA studbook data. The mean (5−95% CI) rate of inbreeding (Δ*F* = 0.0615 [5−95% CI, 0.0239–0.1001]) per generation was calculated by comparing the genome‐wide heterozygosity of 20 parents and their 44 offspring in the IAA. The level of inbreeding in 2020 was calculated using *F* = 1 – (1 – Δ*F*)*^t^*, where *t* is the number of generations since 1995. The generation time was assumed to be 5.6 years. Due to the increase in inbreeding coefficient between 1995 (white bars) and 2020 (gray bars), extinction probability of the population also increased.

### Conservation dependency

**FIGURE 7:**
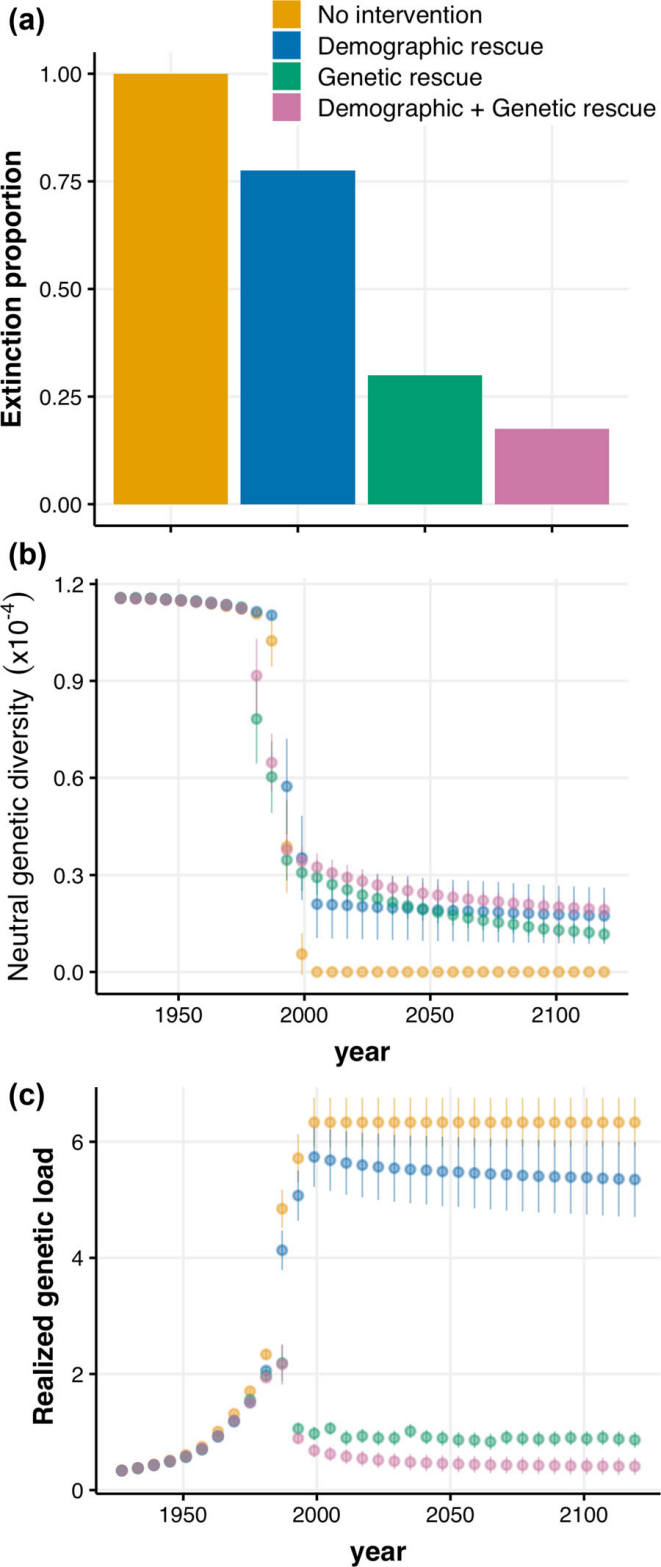
(a) Proportion of replicates that went extinct by year 2120. (b‐c) Mean (dots) and standard deviation (bars) of (b) nucleotide diversity and (c) fitness effect of the genetic load (i.e., realized load; Bertorelle et al., 2022) across replicates in genomic simulations with SLiM (Haller & Messer, 2019) of the free‐living pink pigeon population. Metrics include the effect of extinct replicates (a) by taking their last recorded values into account for the mean and standard deviation calculations in subsequent steps. The simulated population experienced a severe bottleneck and was subjected to different management scenarios: no intervention (yellow), demographic rescue (blue), genetic rescue (green) and demographic + genetic rescue (pink) (see Appendix S4 for details).

The SLiM simulations showed that the counterfactual scenario (no population intervention) resulted in 100% extinction before 2120, shortly after the bottleneck (Figure 7). The demographic scenario also resulted in a high rate of extinction; in ∼75% of all runs, birds became extinct by 2000. Both scenarios had a high realized load, which caused populations to collapse. In contrast, both genetic rescue scenarios successfully reduced the realized load and extinction probability. Populations with genetic rescue but without population intervention went extinct in 30% of all runs, and this scenario resulted in considerable erosion of genetic neutral variation (Figure 7). The best scenario was a combination of genetic rescue and demographic rescue, corroborating the Vortex results. This scenario resembled the conservation rescue of the pink pigeon most closely. Although it too resulted in some extinctions (17.5%) and a drop in nucleotide variation compared with the ancestral population, the realized load returned (almost) to its prebottleneck state (Figure 7).

## DISCUSSION

Four decades of intensive conservation management have increased the census population size of the free‐living pink pigeon metapopulation in Mauritius from 12 to over 400 birds. Consequently, the species has been downlisted twice on the IUCN Red List. However, we found that the metapopulation continues to lose genetic variation at an alarming rate. This conclusion is supported by the population genetic analysis of both RAD‐sequencing and microsatellite data. In addition, an analysis of longevity data of 1112 birds in zoos collected over 4 decades revealed a high genetic load of 15 LEs for this trait. Vortex simulations indicated that without renewed genetic rescue, the free‐living population in Mauritius is likely to become extinct within the next 100 years. However, given the results of our sensitivity analysis and uncertainty about some parameter settings, the Vortex results should be interpreted with caution. Nevertheless, we can be relatively confident that extinction is likely if supplementation is stopped altogether (i.e., no demographic or genetic rescue). Furthermore, computer simulations indicated that genetic rescue from European and U.S. zoo populations could elevate the genetic variation. Computer simulations in SLiM (Haller & Messer, 2019) indicated that such renewed conservation action could also reduce the genetic load of expressed deleterious mutations (i.e., the realized load) (van Oosterhout, 2020; Mathur & DeWoody, 2021; Bertorelle et al., 2022) and improve the long‐term viability of the species.

Inbreeding is difficult to avoid after a severe bottleneck and inevitably leads to genomic erosion (Frankham, 2015; Hedrick & Garcia‐Dorado, 2016; Gilroy et al., 2017; Díez‐del‐Molino et al., 2018; Kardos et al., 2018; Bortoluzzi et al., 2020). Some breeding pairs may be genetically incompatible, for example, parents with high mean kinship or with many recessive deleterious mutations at the same genetic loci. Such pairs may have failed to reproduce viable offspring, thus reducing *N*_e_. Furthermore, early‐released birds appear to have contributed more to the gene pool than birds that were released later in the recovery program (Swinnerton et al., 2004). Such priority effect is well known (e.g., Monopolization Hypothesis, De Meester et al., 2002), but may be particularly pronounced in long‐lived species that maintain breeding territories. Initial founder birds would have been able to establish themselves in the best territories, preventing newly arriving birds from breeding. Postreproductive survival of females may have further exacerbated this skew in reproductive success; reproductively inactive pairs continued to occupy valuable breeding territories, preventing other birds from reproducing. Altogether, this could explain the significant variance in reproductive success, with a large proportion of chicks being produced by a few successfully breeding pairs (Swinnerton et al., 2004). Eventually, this process could have reduced the *N*_e_ and accelerated loss of genetic variation.

Due to its large ancestral population size, the genome of the pink pigeon has accumulated a high genetic load of deleterious mutations. We estimated that the zoo population possesses circa 15 LEs for longevity (adult survival). Given that the birds in this ex situ gene pool are closely related to individuals in the free‐living metapopulation, we assume this is a reasonable approximation of the genetic load in the wild population. The species’ load for longevity is slightly higher than the average of 12 diploid LEs found across the life history of bird and mammal species (O'Grady et al., 2006). Furthermore, its load is comparable to in 2 other bottlenecked bird populations: 10–15 LEs acting on chick and juvenile survival in the bottlenecked little spotted kiwi (*Apteryx owenii*) (Taylor et al., 2017) and 14 LEs for survival until fledging in the New Zealand hihi (*Notiomystis cincta*) (Brekke et al., 2010). A high genetic load poses a long‐term threat to the viability of bottlenecked species. Genomics‐informed conservation could help minimize genetic load in the future, during ex situ breeding and reintroduction programs (van Oosterhout, 2020), and thus improve long‐term viability.

Species with a large ancestral population size tend to possess a high “masked load” of recessive deleterious mutations that have remained largely hidden from selection when the population size was large. This masked load is the part of the genetic load that causes inbreeding depression during inbreeding, and it is also known as the “inbreeding load” (Crow, 1970), or “potential load” (Mathur & DeWoody, 2021). Our SLiM simulations (Haller & Messer, 2019) showed that recent and current inbreeding converted this masked load into a realized load (Bertorelle et al., 2022). These simulations also quantified the benefit of genetic rescue, illustrating the positive impact of a sustained release of captive‐bred individuals from the GDEWS. Genetic rescue offered a 2‐fold benefit: it has helped reduce the loss of genetic diversity, and it has minimized the realized load, thereby reducing the severity of inbreeding depression. Our results highlight that genetic rescue must be accompanied by demographic recovery for natural selection to be effective, enabling the purging of the realized load. The simulations indicated that reintroductions may need to continue to ensure long‐term genetic benefits. Genetic rescue programs might not be effective as a one‐off solution to counteract genetic erosion. If only a few individuals are translocated, or if there is no demographic rescue, the benefits of genetic rescue may be very short lived. Such negative effects of single genetic‐rescue events have been observed in Isle Royale wolves (*Canis lupus*) (Hedrick et al., 2019), Artic foxes (*Vulpes lagopus*) (Lotsander et al., 2021), and simulation studies (Kyriazis et al., 2021).

In the case of the pink pigeon, there are further limitations to the benefits that genetic rescue can offer. The ex situ population at the GDEWS was founded by 12 individuals taken from 1 free‐living population from 1976 to 1981; consequently, its gene pool has been subjected to considerable genetic drift. However, given that we detected novel allelic microsatellite variation in zoos, the genetic diversity in the GDEWS can be enhanced by introducing birds from zoos. Conserving genome‐wide genetic variation is considered the best approach to prevent inbreeding depression and reduce the extinction risk (Kardos et al. 2021), and hence, increasing the diversity in the GDEWS could improve the effectiveness of genetic rescue in the future. However, the risks and benefits of such conservation action should be carefully considered for each species (e.g., Hohenlohe et al., 2019; Bell et al., 2019; Ralls et al., 2020; Teixeira & Huber, 2021; Robinson et al., 2021).

Our results indicated that the pink pigeon is still at a considerable risk of extinction. Although this is reflected in its most recent IUCN Red List assessment that classified the species as “vulnerable,” this evaluation does not highlight the high conservation dependency of the species. Criteria A–D of the IUCN Red List assessment cannot detect all possible threats, particularly to populations that are well managed and benefitted (demographically) from releases of captive‐bred individuals. By focusing on population size, changes in demography, habitat fragmentation, and geographic range size (IUCN criteria A–D) and not explicitly assessing genomic erosion, the IUCN potentially underestimates the extinction risk in such cases. Evidence of genomic erosion can be used in quantitative analyses of extinction probability (IUCN assessment criterion E), but unfortunately, this criterion is rarely used when evaluating the extinction risk of birds (Appendix S3k) (Collen et al., 2016). Until recently, there have been relatively few species for which genomic data are available that would allow robust quantitative analyses.

Nevertheless, we do not call for a reassessment or uplisting of the IUCN Red List status of the pink pigeon. Considerable resources have been dedicated to enable the analyses summarized in this study, and this is only feasible for few threatened species. Given that the IUCN assessment criteria must remain similar across species (and over time), one should not apply scientific tools with higher sensitivity for just a small subset of species. Increasingly, calls are made to integrate genetics in IUCN assessment (e.g., Garner et al. 2020). However, unless these techniques can be applied ubiquitously, it risks biasing the sensitivity of the assessment in favor of a few flagship species for which such analyses are feasible, undermining the comparability of entries on the IUCN Red List. Genomic analyses can significantly enhance conservation assessments for many threatened species, and such data must be incorporated in a set of standardized metrics to help guide conservation when possible. Crucially, these metrics need to be uncomplicated so that they can be understood and applied by the entire conservation community (van Oosterhout, 2021).

We propose that the recently developed IUCN Green Status of Species (Akçakaya et al., 2018; IUCN, 2021; Grace et al., 2021), a new component of the IUCN assessments, might offer a better platform for use of modeling and genomic data to inform the assessment of species conservation status. The IUCN Green Status of Species is determined based on a species’ recovery and conservation impact (Akçakaya et al., 2018; IUCN, 2021; Grace et al., 2021), and recovery is assigned a score (i.e., green score) that quantifies a species’ viability and functionality. Given that the IUCN Green Status assessment is novel and still under development, changes to its assessment protocol are more easily incorporated. As such, it can readily accommodate insights from modern genomic analyses, bioinformatics, and computer models. These are more sensitive than the genetic analyses available in 1994 and 2000, at the time that the current set of IUCN Red List rules were adopted and modified (Mace et al., 2008). Because only 181 species have been evaluated preliminarily with the green‐status protocol (Grace et al., 2021), compared with ∼150,000 species on the IUCN Red List, the inclusion of genomic data is a more feasible proposition. Most importantly, IUCN Green Status assessment is an indicator of progress toward species’ recovery and ecological functionality (Akçakaya et al., 2018), and the genomic health of the individuals and populations forms part of that journey toward full recovery.

## Supporting information

Appendix A1

Appendix A2

Appendix A3

Appendix A4
