# Supplementary material for: Genomic erosion in a demographically recovered bird species during conservation rescue
Source: Conserv Biol. 2022 May 12;36(4):e13918. doi: 10.1111/cobi.13918 (PMC9546124; doi:10.1111/cobi.13918)
Supplement: Supplementary file 1 — Appendix A1 [file COBI-36-0-s003.docx]

**Appendix S1a: Genome Assembly**

Five grams of frozen leg muscle was ground in liquid nitrogen before proteinase K digestion followed by Phenol:Chloroform:Isoamyl Alcohol precipitation and then RNA digestion using RNAseI. QC was performed using the Qubit dsDNA HS Assay Kit (Life Technologies). A PCR-free library was prepared from approximately 0.5 μg of DNA, in keeping with the input requirements of DISCOVAR de novo (Weisenfeld et al. 2014). Jumping or mate pair (sizes 4, 8, 12 Kb) were produced from 2, 5, 8 ug of DNA using the Nextera Mate Pair Library Prep Kit, post fragmentation reaction DNA size selection used a Sage Science BluePippin. Fragment ends were repaired with biotin labelled nucleotides, circularised then randomly sheared using a Covaris S2 acoustic sonicator (Covaris Inc.) The sheared fragments were then bound to M280 streptavidin-coated beads (Thermo Fisher). The biotin containing junction fragments were end repaired and A-tailed before the addition of Illumina TruSeq adapters, followed by 16 cycles of PCR to enrich the final libraries (Illumina) and purification using AMPure XP beads (Agencourt). All libraries were QC checked with the Bioanalyzer DNA HS assay system (Agilent Technologies Inc.) and quantified by both Qubit dsDNA HS Assay Kit and qPCR using a KAPA Library Quantification Kit on a StepOnePlus Real-Time PCR System (Life Technologies). Illumina sequencing was performed using paired-end sequencing (2 x 251 bp), with a 1% PhiX spike, on an Illumina HiSeq 2500 sequencer in rapid-run mode. Following sequencing, read quality was assessed with FastQC (Andrews 2010). The draft genome was assembled from paired end data using DISCOVAR de novo (Weisenfeld et al. 2014) to generate contigs. Mate pair libraries were assessed and filtered for the presence of the junction adaptor in one or both reads using NextClip (Leggett et al. 2014) before scaffolding the contigs using SOAPdenovo (Li et al. 2010). Analysis of the draft pink pigeon genome assembly using Abyss-fac (Simpson et al. 2009) which revealed the N50 to be 8 Mbp. An assessment of the completeness of the assembled genome was calculated using BUSCO v. 3.1.0 (Waterhouse et al. 2018) and the aves database (aves_odb9). A total of 4915 BUSCO groups were searched and of those 4639 (94.38%) were identified in the genome assembly. There were 4586 (93%) BUSCO groups identified as single copy and complete, 168 (3.42%) as fragmented and 108 (2.20%) were missing.

A KAT kmer-spectra copy number plot (S1a), which compares the kmers found in a set of raw reads to those found in the final assembled genome, was produced using KATv2.3.4 (Mapleson et al. 2017) with a kmer length of 31. This gives a measure of the quality of the genome when quality is thought of as the correct motifs, the correct number of times, in the correct order. This plot confirmed that the final scaffolded assembly had captured most the information present in the reads and that there were no obvious misassembly errors – represented by kmers present in the assembly and not in the reads.


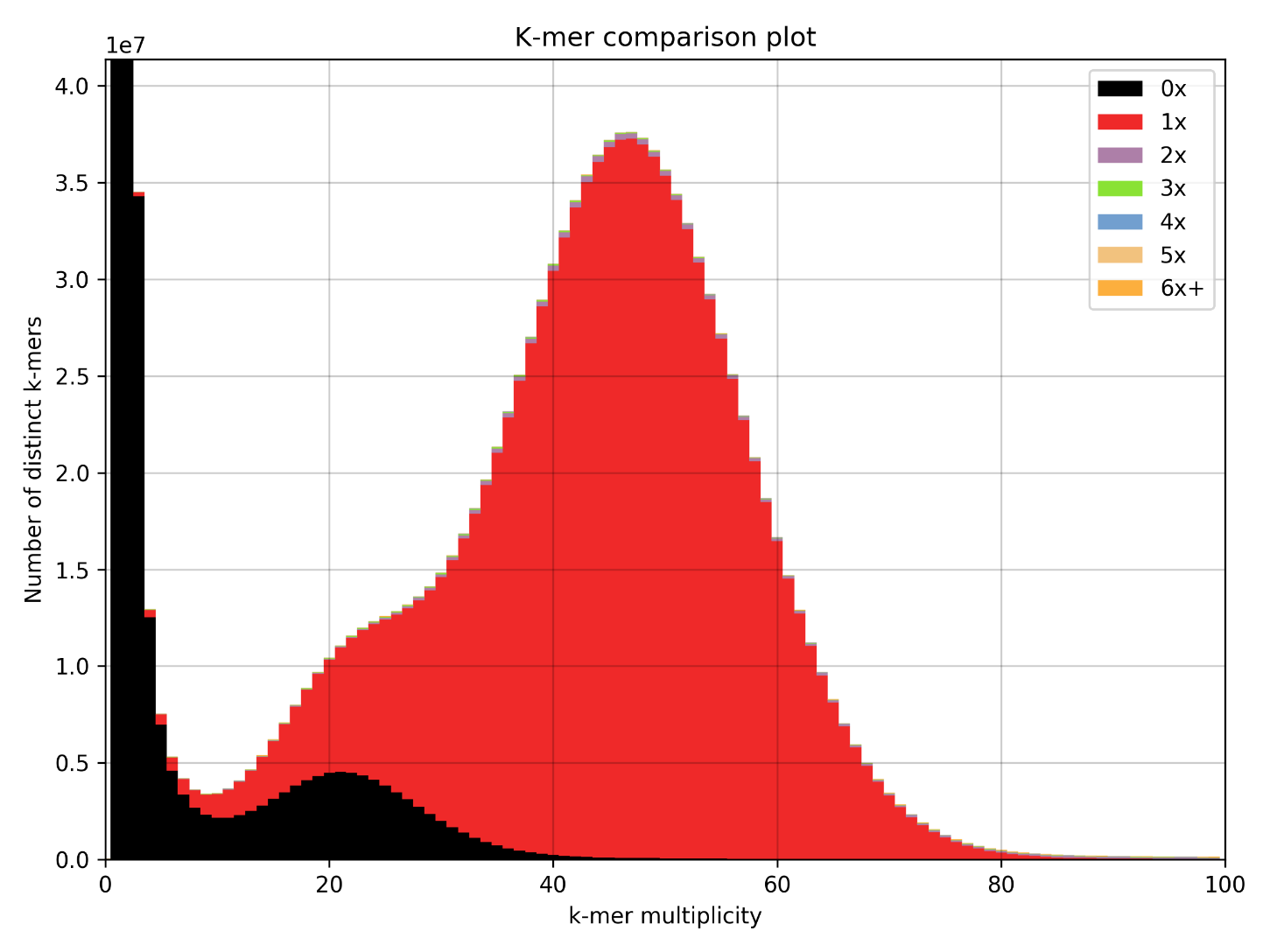


**Appendix S1b.** KAT (Mapleson et al. 2017) k-mer spectra copy number plot is a way of visualising that the k-mers found in the reads appear in the genome the correct or expected number of times. Plots are coloured to show how many times k-mers from the reads appear in the assembly; frequency of occurrence (x-axis) and number of distinct k-mers (y-axis). The plot was generated with k =31. The black peak on the left represent likely errors present in the reads at low numbers but not included in the assembly. Completeness appears good with little missing content and no content in the genome that is not present in the reads - the assembler has not created content.

**Appendix S1c:** *Pseudomolecule and runs of homozygosity generation*

The pink pigeon scaffolds were super-scaffolded into pseudochromosomal sequences using RaGOO v1.1 (Alonge et al. 2019) together with MiniMap2 v2.17 (Li 2018) aligning to the genome of the zebra finch, *Taeniopygia guttata*. The command line used was: “ragoo.py -t 10 -g 100 -s”. A custom Python3 script, mapper.py, was then used to transfer the VCF coordinates. VCFtools v0.1.16 (Danecek et al. 2011) was then used to calculate the allele frequency at each genotyped site. BCFtools from the HTSlib suite was then used to calculate ROH with the following command line:

“bcftools roh -G 30 -O rz --AF-file”

A custom Python3 script (calculate_froh.py) was then used to calculate the Froh for each individual. Froh was calculated as the proportion of sequence length estimated to be in ROH over the total length of autosomal chromosomes. VCFtools (Danecek et al. 2011) was then used to transform the mapped VCF into a 012-format matrix file. LoD intervals were calculated using an adapted version of the “lodRohDetection.R” script (Kardos et al. 2018). We used windows of 100 markers, with a step size of 10, and a minimum number of 50 SNPs per window. The matrices created by the script were used to plot the loss of heterozygosity in the chromosomes, reimplementing the same procedure by Kardos et al. (2018) in a custom Python3 Jupyter notebook, using Pandas and Matplotlib (Hunter 2007).

**Literature cited**

Alonge M, Soyk S, Ramakrishnan S, Wang X, Goodwin S, Sedlazeck FJ, Lippman ZB, Schatz MC. 2019. RaGOO: fast and accurate reference-guided scaffolding of draft genomes. Genome Biology DOI: 10.1186/s13059-019-1829-6.

Andrews S. 2010. FastQC: A Quality Control Tool for High Throughput Sequence Data. Babraham Babraham Institute, Cambridge. Available from https://www.bioinformatics.babraham.ac.uk/projects/fastqc/ (accessed December 2013).

Danecek P, et al. 2011. The variant call format and VCFtools. Bioinformatics DOI: 10.1093/bioinformatics/btr330.

Hunter JD. 2007. Matplotlib: A 2D graphics environment. Computing in Science and Engineering DOI: 10.1109/MCSE.2007.55.

Kardos M, Åkesson M, Fountain T, Flagstad Ø, Liberg O, Olason P, Sand H, Wabakken P, Wikenros C, Ellegren H. 2018. Genomic consequences of intensive inbreeding in an isolated wolf population. Nature Ecology and Evolution DOI: 10.1038/s41559-017-0375-4.

Leggett RM, Clavijo BJ, Clissold L, Clark MD, Caccamo M. 2014. NextClip: an analysis and read preparation tool for Nextera Long Mate Pair libraries. Bioinformatics DOI: 10.1093/bioinformatics/btt702.

Li R, et al. 2010. The sequence and de novo assembly of the giant panda genome. Nature DOI: 10.1038/nature08696.

Li H. 2018. Minimap2: pairwise alignment for nucleotide sequences. Bioinformatics DOI: 10.1093/bioinformatics/bty191.

Mapleson D, Garcia Accinelli G, Kettleborough G, Wright J, Clavijo BJ. 2017. KAT: a K-mer analysis toolkit to quality control NGS datasets and genome assemblies. Bioinformatics DOI: 10.1093/bioinformatics/btw663.

Simpson JT, Wong K, Jackman SD, Schein JE, Jones SJM, Birol I. 2009. ABySS: a parallel assembler for short read sequence data. Genome Research DOI: 10.1101/gr.089532.108.

Waterhouse RM, Seppey M, Simão FA, Manni M, Ioannidis P, Klioutchnikov G, Kriventseva EV, Zdobnov EM. 2018. BUSCO applications from quality assessments to gene prediction and phylogenomics. Molecular biology and evolution DOI: 10.1093/molbev/msx319.

Weisenfeld NI, et al. 2014. Comprehensive variation discovery in single human genomes. Nature Genetics DOI: 10.1038/ng.3121.
