# Supplementary material for: Genomic erosion in a demographically recovered bird species during conservation rescue
Source: Conserv Biol. 2022 May 12;36(4):e13918. doi: 10.1111/cobi.13918 (PMC9546124; doi:10.1111/cobi.13918)
Supplement: Supplementary file 2 — Appendix A2 [file COBI-36-0-s001.docx]

**Appendix S2: Microsatellite analysis**

**Appendix S2a.** Summary details of the 22 microsatellite loci genotyped for the Pink Pigeon.

| Locus | Primer sequences (5'-3') | No. alleles | Obs. allele sizes | H_e_ |
| --- | --- | --- | --- | --- |
| **Nma25A05** | F: CTTAAAGGTATTTTCTGACTGAAATG | 10 | 166-221 | 0.812 |
|  | R: TCGAGGGTCAAAATCATTAGA |  |  |  |
| **Nma25C04** | F: AGCTTCCTGAAGGCAACATC | 5 | 190-214 | 0.636 |
|  | R: GCCTGCTCTCCATGACTTG |  |  |  |
| **Nma25F10** | F: TGCTGCTACAGACCATCCTG | 3 | 99-102 | 0.454 |
|  | R: AGGTCAATACCATGGGAGAGG |  |  |  |
| **Nma26A03** | F: TTGTAGAGGGCAAACACAAGG | 5 | 169-185 | 0.601 |
|  | R: TGGTTGGAAGAGACCCTCAG |  |  |  |
| **Nma26A08** | F: CTAGCAAGGCTGGTATGCTTG | 5 | 92-117 | 0.583 |
|  | R: CTTCTGAATACCTTCTGCCTGTG |  |  |  |
| **Nma26C08** | F: CAGGAGAATTGCAAAGAATGG | 4 | 189-200 | 0.598 |
|  | R: TCCAGAATGAGCATTATTTATCTCC |  |  |  |
| **Nma26C09** | F: TGGTATCACACTTTCAGGAATCAC | 5 | 153-169 | 0.788 |
|  | R: TCTGCATTGGTCTGACAGAAAG |  |  |  |
| **Nma26D12** | F: GATCCATTTACGATGTCCCTTC | 4 | 174-186 | 0.669 |
|  | R: GGAAAGTCTTCTCAGGTTCAGG |  |  |  |
| **Nma29A10** | F: ACAGTTGAGTGCCTGGTTGTC | 5 | 183-197 | 0.736 |
|  | R: CACCTTGGTGCTTCTCTCATAAC |  |  |  |
| **Nma29A11** | F: TTGATTCAGGTCACTGTGTGG | 6 | 130-165 | 0.66 |
|  | R: AGAAGAACGCTCTCCTGCTG |  |  |  |
| **Nma29B08** | F: TTTGTTGTTGCTTGGTTTGG | 2 | 159, 161 | 0.483 |
|  | R: AGAAACAGAGCTGACACAGCAG |  |  |  |
| **Nma29F05** | F: TATGAGGATTCTGCGAACAGG | 6 | 95-107 | 0.508 |
|  | R: ACATGCTCTGGTTGAGAATTAGC |  |  |  |
| **Nma30A05** | F: TCAACTATTCAACCCAGAATACCC | 3 | 145-156 | 0.51 |
|  | R: TGCCCTTAAACACCTGTATGC |  |  |  |
| **Nma30B11** | F: AAGGCTGGGAGTAGCAAACAC | 2 | 223, 226 | 0.082 |
|  | R: TCTGCCAGTTTGTATCAGCAAG |  |  |  |
| **Nma30C05** | F: TCTTCTTTAACAGTTGGTCCATAGC | 9 | 153-176 | 0.754 |
|  | R: TCTTCCCTAAAGAGGCAGGAC |  |  |  |
| **Nma30D01** | F: GGTGGGTTTATTCTGCTTTCC | 7 | 118-139 | 0.762 |
|  | R: GTTGTAACGGGACATAACAAAGC |  |  |  |
| **Nma31A06** | F: AGTTTCTGGCGGCTACATTC | 3 | 122-130 | 0.223 |
|  | R: ACAGGTGTTGAAATCACACAGAC |  |  |  |
| **Nma31A10** | F: CAAACTCACAGCTAACCATTGTG | 3 | 168-172 | 0.223 |
|  | R: CCTGCCCACTCTGCTAGTTC |  |  |  |
| **Nma31B08** | F: TGTTCCTCCAGTAACCAGCTC | 4 | 105-113 | 0.668 |
|  | R: AGTTGGACCAGAAATGTGTACG |  |  |  |
| **Nma31C11** | F: TGTTGCATGCAGGGTCTG | 3 | 207-211 | 0.121 |
|  | R: TTTGGAATCCTTAAATGAGAAATCAC |  |  |  |
| **Nma31D06** | F: TTCTCAGTTCCCATCCCATC | 4 | 179-188 | 0.564 |
|  | R: AACTCCTACCTGACAGGGACAC |  |  |  |
| **Nma31F11** | F: TGCGAGCTTCTCTGTTATTGG | 4 | 162-169 | 0.223 |
|  | R: TCACTGCTGCTTCACAAGATG |  |  |  |

*STRUCTURE analysis using microsatellite data*

A Bayesian clustering approach was used to detect the most likely number of genetic clusters (K) among captive and free-living subpopulations over the two sampling periods (1990s and 2000s) using STRUCTURE v2.3.4 (Pritchard et al. 2000). Ten repeated runs were completed with K ranging from one to 15, for 500,000 MCMC iterations with a burn-in of 100,000 under the admixture model with correlated allele frequencies (Falush et al. 2003). Prior information was provided (population/temporal information) to assist clustering (Hubisz et al. 2009). Two approaches were used to identify the most likely number of clusters in Structure Selector (Li & Liu 2018), firstly assignment values, log likelihood scores and delta K were evaluated using the Evanno method (Evanno et al. 2005). Secondly, as this method has been shown to underestimate clusters in cases where sampling of subpopulations is uneven (Janes et al. 2017), we calculated estimators based on a count of the number of independent clusters from user defined groups (Puechmaille 2016) which have shown to be more accurate than traditional delta K and log likelihood methods. Clusters were plotted using in Structure Selector (Li & Liu 2018).

**Appendix S2b**. Delta K plot using the Evanno method (Evanno et al. 2005) for STRUCTURE (Pritchard et al. 2000) analysis obtained from STRUCTURE SELECTOR (Li & Liu 2018), on all free-living and captive samples for K=1-15. The value of K that has the highest delta value is considered the most likely number of K.

**Appendix S2c**. Estimators of K accounting for uneven sample size (Puechmaille 2016) obtained from STRUCTURE SELECTOR (Li & Liu 2018), on all free-living and captive samples for K=1-15. The value of K that has the highest delta value is considered the most likely number of K, here the most likely number of clusters is K=7.


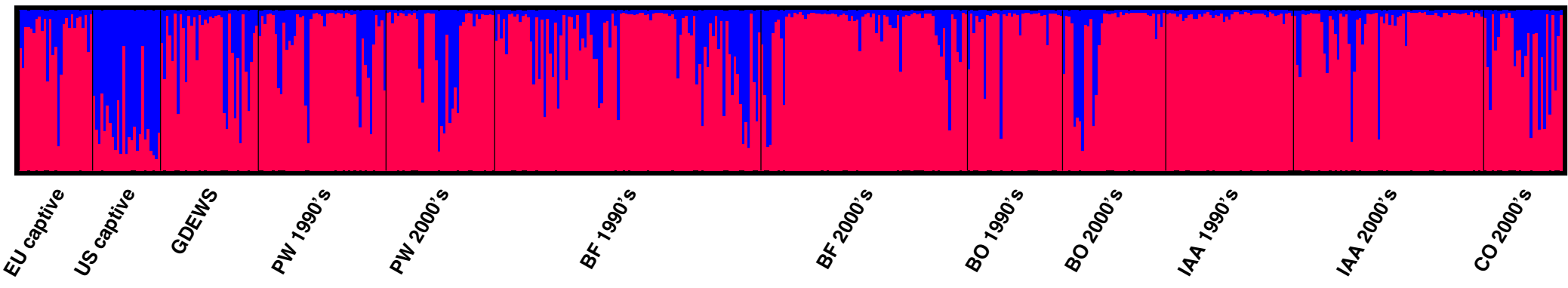


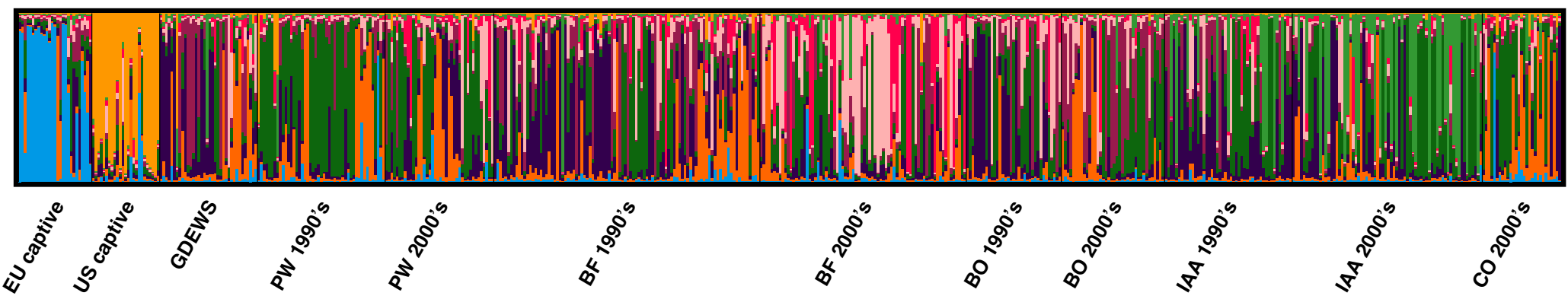
**Appendix S2d**. STRUCTURE cluster plots for K=2 (top) and K=9 (bottom).

**Appendix S2e**. Effective population sizes (*N_e_*) and 95% confidence intervals, using the linkage disequilibrium method (*Pcrit* = 0.02) for the free-living metapopulation in 1993 and 2010, and each free-living subpopulation for the two sampled groups 1990s (1993–1997) and 2000s (2007–2011).

|  | **N** | **LDM N_e_** | **95% CIs** | |
| --- | --- | --- | --- | --- |
|  |  |  |  |  |
| **Free-living metapopulation** |  |  |  |  |
| 1993 | 40 | 52.5 | 36.2 | 88.1 |
| 2010 | 64 | 49.4 | 40.8 | 61.1 |
| **Pigeon Wood** |  |  |  |  |
| 1990s | 47 | 22.5 | 19.3 | 26.5 |
| 2000s | 40 | 22.3 | 18.4 | 27.5 |
| **Plaine Lievre** |  |  |  |  |
| 1990s | 98 | 67.3 | 56.5 | 81.9 |
| 2000s | 76 | 40.3 | 34.7 | 47.2 |
| **Bel Ombre** |  |  |  |  |
| 1990s | 35 | 29.5 | 21.6 | 43.0 |
| 2000s | 38 | 23.7 | 19.6 | 29.9 |
| **Ile aux Aigrettes** |  |  |  |  |
| 1990s | 47 | 22.7 | 18.9 | 28.0 |
| 2000s | 70 | 23.3 | 20.3 | 26.8 |

**Appendix S2f.** Effective population size (*N_e_*) estimates of the free-living pink pigeon subpopulations sampled between 1994 and 2008 based on an analysis of 4000 independent SNPs and 100 replicates in the software NeEstimator (Do et al. 2014). Shown are the sample sizes used, the mean effective population size (*N_e_*), the standard deviation (SD), and the 5 and 95% CI.

| Population | Sample size | Mean *N_e_* | SD | 5% CI | 95% CI |
| --- | --- | --- | --- | --- | --- |
| Bel Ombre | 12 | 6.62 | 0.37 | 5.87 | 7.36 |
| Plaine Lievre | 12 | 5.51 | 0.22 | 5.08 | 5.95 |
| Combo | 25 | 5.08 | 0.16 | 4.76 | 5.4 |
| Ile aux Aigrettes | 116 | 4.53 | 0.13 | 4.27 | 4.79 |
| Pigeon Wood | 10 | 5.82 | 1.19 | 3.43 | 8.2 |

**Appendix S2g. (a)** Temporal trends in mean heterozygosity ±SE (H_e_; black circles) and allelic richness ±SE (Ar; red squares), for each subpopulation. **(b)** Analysis with the software STRUCTURE (Pritchard et al, 2000) identified seven groups. **(c)** Effective population sizes (*N_e_*) and 95% CI, using the linkage disequilibrium method (*Pcrit* = 0.02) for the free living metapopulation in 1993 and 2010, and each free-living subpopulation for the two sampled groups 1993–1997 (1990s) versus 2007–2011 (2000s). **(d)** Estimates of gene flow between captive and free-living subpopulations both sampling groups; 1990s/2000s. Arrows represent direction of migration rates. Gerald Durrell Endemic Wildlife Sanctuary (GDEWS) migration is calculated for the recovering 1990s time-period only, and for Combo (CO) rates are calculated for the recent 2000s time-period only. Significant rates of gene flow (Nm>0.10) are in bold. **(e)** The effect of genetic rescue with zoo birds on the mean (±StDev) number alleles at 22 loci on the gene pool of the combined individuals sampled from the free-living subpopulations in 2010 (n=64 birds sampled in 2010).


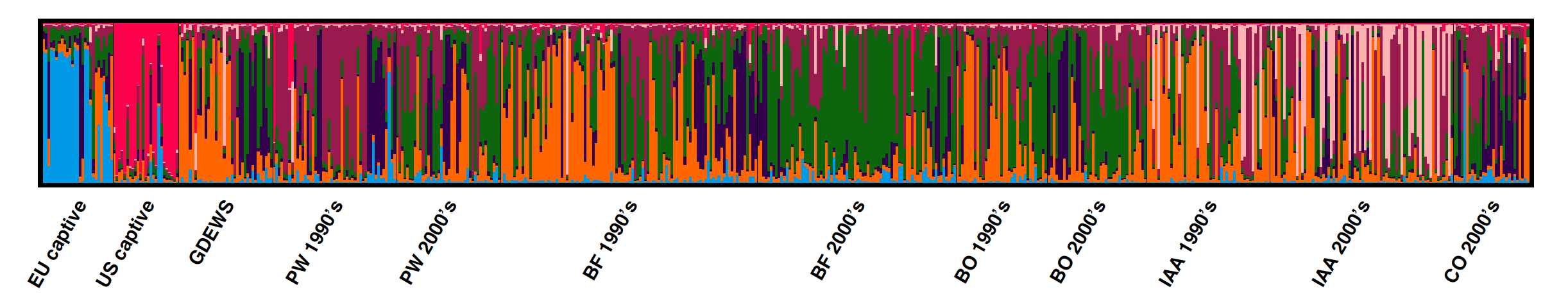


**a**.

**b.**

**c.**

**d**.

**e**.

*Temporal patterns of gene-flow*

Gene flow (recent migration rates measured by the proportion of the population comprising migrants = *Nm*) between subpopulations was calculated for the 1990s and 2000s time periods using a Bayesian approach in BayesAss^9^. Three independent runs were conducted using different starting seeds to ensure consistency, with 50,000,000 iterations including a burn-in of 5,000,000 and sampling every 100^th^ iteration with adjusted chain delta values at 0.2 for the 1990s dataset and 0.3 for the 2000s dataset. Convergence was assessed using Tracer^10^ and 95% confidence intervals (CI) were calculated for each migration rate.

**Appendix S2h**. Genetic differentiation between *in-situ* and *ex-situ* captive pink pigeons and free-living subpopulations of pink pigeons on Mauritius separated into the time periods, the 1990s and 2000s. F_ST_ is below the diagonal, D_JOST_ is given above the diagonal. All pairwise values are significantly different (*p*<0.05).

|  | *Ex-situ*  captive | | *In-situ* captive | **Free living subpopulations on Mauritius** | | | | | | | | |
| --- | --- | --- | --- | --- | --- | --- | --- | --- | --- | --- | --- | --- |
|  | **EU**  **cap** | **US**  **cap** | **GDEWS**  **cap** | **PW 1990s** | **PW 2000s** | **BF 1990s** | **BF 2000s** | **BO 1990s** | **BO 2000s** | **IAA 1990s** | **IAA 2000s** | **CO 2000s** |
| **EU cap** | - | 0.181 | 0.104 | 0.103 | 0.097 | 0.066 | 0.068 | 0.090 | 0.095 | 0.103 | 0.112 | 0.089 |
| **US cap** | 0.078 | - | 0.143 | 0.184 | 0.153 | 0.149 | 0.174 | 0.159 | 0.165 | 0.167 | 0.186 | 0.141 |
| **GDEWS** | 0.050 | 0.058 | - | 0.078 | 0.037 | 0.027 | 0.046 | 0.046 | 0.040 | 0.039 | 0.056 | 0.058 |
| **PW 1990s** | 0.050 | 0.071 | 0.036 | - | 0.056 | 0.040 | 0.059 | 0.050 | 0.056 | 0.042 | 0.067 | 0.057 |
| **PW 2000s** | 0.049 | 0.063 | 0.022 | 0.028 | - | 0.034 | 0.034 | 0.030 | 0.045 | 0.039 | 0.039 | 0.023 |
| **BF 1990s** | 0.032 | 0.056 | 0.015 | 0.019 | 0.018 | - | 0.020 | 0.017 | 0.033 | 0.024 | 0.050 | 0.059 |
| **BF 2000s** | 0.037 | 0.071 | 0.024 | 0.029 | 0.019 | 0.011 | - | 0.033 | 0.031 | 0.042 | 0.050 | 0.059 |
| **BO 1990s** | 0.046 | 0.064 | 0.025 | 0.026 | 0.019 | 0.012 | 0.020 | - | 0.031 | 0.032 | 0.051 | 0.042 |
| **BO 2000s** | 0.047 | 0.066 | 0.023 | 0.028 | 0.025 | 0.017 | 0.018 | 0.020 | - | 0.048 | 0.058 | 0.054 |
| **IAA 1990s** | 0.050 | 0.065 | 0.022 | 0.022 | 0.021 | 0.013 | 0.022 | 0.019 | 0.025 | - | 0.024 | 0.048 |
| **IAA 2000s** | 0.055 | 0.074 | 0.028 | 0.032 | 0.022 | 0.022 | 0.025 | 0.027 | 0.029 | 0.015 | - | 0.048 |
| **CO 2000s** | 0.046 | 0.058 | 0.030 | 0.029 | 0.017 | 0.022 | 0.030 | 0.024 | 0.028 | 0.026 | 0.026 | - |

**Literature cited**

Do, C., Waples, R. S., Peel, D., Macbeth, G. M., Tillett, B. J., Ovenden, J. R. (2014). NeEstimator v2: re-implementation of software for the estimation of contemporary effective population size (Ne) from genetic data. *Molecular Ecology Resources* DOI:10.1111/1755-0998.12157.

Evanno, G., Regnaut, S., Goudet, J. (2005). Detecting the number of clusters of individuals using the software STRUCTURE: a simulation study. *Molecular Ecology* DOI:10.1111/j.1365-294X.2005.02553.x.

Falush, D., Stephens, M. & Pritchard, J. K. (2003). Inference of population structure using multilocus genotype data: linked loci and correlated allele frequencies. *Genetics* 164:1567-1587.

Hubisz, M. J., Falush, D., Stephens, M., & Pritchard, J. K. (2009). Inferring weak population structure with the assistance of sample group information. *Molecular Ecology Resources* 9:1322-1332.

Janes, J. K., Miller, J. M., Dupuis, J. R., Malenfant, R. M., Gorrell, J. C., Cullingham, C. I., & Andrew, R. L. (2017). The K= 2 conundrum. *Molecular Ecology*, *26*(14), 3594-3602. DOI:10.1111/mec.14187

Li, Y. L., & Liu, J. X. (2018). StructureSelector: A web‐based software to select and visualize the optimal number of clusters using multiple methods. *Molecular Ecology Resources*, *18*(1), 176-177. DOI:10.1111/1755-0998.12719.

Pritchard, J. K., Stephens, M., & Donnelly, P. (2000). Inference of population structure using multilocus genotype data. *Genetics*, 155, 945-959.

Puechmaille, S. J. (2016). The program structure does not reliably recover the correct population structure when sampling is uneven: subsampling and new estimators alleviate the problem. *Molecular Ecology Resources*, *16*(3), 608-627.
