# Supplementary material for: Genomic erosion in a demographically recovered bird species during conservation rescue
Source: Conserv Biol. 2022 May 12;36(4):e13918. doi: 10.1111/cobi.13918 (PMC9546124; doi:10.1111/cobi.13918)
Supplement: Supplementary file 3 — Appendix A3 [file COBI-36-0-s002.docx]

**Appendix S3: RAD-seq and Vortex simulations**

*Generation of RAD-seq data.*

We removed a 10 ul of blood clot from ethanol storage. A TE buffer was to this sample added prior to the addition of a modified high detergent buffer (6.7% SDS and 7.1% Triton-X-100), which decreases inhibitor Y HAEM levels in the final DNA, followed by the manufacturers protocol (Agencourt® GenFind™ V2 Blood & Serum Genomic DNA Isolation Kit). The isolated genomic DNA (0.5–1 µg) was restriction digested using high fidelity HF-SbfI (New England Biolabs) before heat-inactivation and P1 Adapter ligation (a modified Illumina adaptor (Baird et al. 2008)). Libraries were then heat-inactivated, pooled (typically 8 libraries per pool) and sheared using a Covaris S2 acoustic sonicator (Covaris Inc.). Fragmented molecules were end repaired and A-tailed before the ligation of the P2 Adapter (another modified Illumina adapter (Baird et al. 2008)), followed by 16 cycles of PCR using Kapa HiFi (Kapa Biosystems) to enrich the final libraries. Libraries were then purified with AMPure XP beads (Agencourt). All libraries were QC checked with the Bioanalyzer DNA HS assay system (Agilent Technologies Inc.) and quantified by both Qubit dsDNA HS Assay Kit and qPCR using a KAPA Library Quantification Kit on a StepOnePlus Real-Time PCR System (Life Technologies). Illumina sequencing was performed using paired-end sequencing (2 x 150 bp), on an Illumina HiSeq 2500 sequencer using a custom Illumina recipe which minimises issues with low complexity sequence (10 Read 1: 14 x dark cycles, 136 x light cycles, Rehyb Read1 sequencing primer, 14 x light cycles; Index read: 8 x light cycles; Read 2: 59 x light cycles) with a 2% PhiX spike, on an Illumina HiSeq 2500 in rapid run mode.

*RAD-seq data processing and population genetic analysis.*

BCL to FASTQ conversion was performed using Illumina’s CASAVA (using base mask y136,y14,y8,y59) then each second read was joined to the start of the corresponding first read to regain the full 150 bp read 1. Reads were demultiplexed using RADplex (Leggett et al. 2013) with a mismatch of 0 followed by read QC using FastQC (Andrews 2010), before adapter removal and quality filtering using the "fastq-mcf" program from the ea-utils package (Aronesty 2011). Reads were further filtered to contain the last 6 bp of the SbfI cut-site (TGCAGG) in the first 6 bases of each read. Reads for each sample were aligned to the scaffolded pink pigeon genome assembly using BWA mem (Li 2013). Alignments were then quality (41) filtered before removing duplicates from the BAM files using SAMtools (Li et al. 2009). SAMtools mpileup and BCFtools (Li 2011) were used to perform the variant calling. VCFtools (Danecek et al. 2011) was used to remove indels and include only SNPs where 96% of the samples had depth >=10 and a minimum combined allele count of 2. An association test using PLINK (Purcell et al. 2007) was performed to identify scaffolds and contigs relating to the sex chromosomes from female/male observation (based on behaviour) data, these sex-associated scaffolds were also filtered from the final VCF as were three pink pigeon individuals where their genotype did not match the pedigree. To investigate relatedness of samples, we constructed a NeighbourNet network using all 43,967 SNPs with SplitsTree v4 (Huson et al. 2008) using K2P distance estimates. SNPs were extracted from the VCF file in fasta format with PGDspider (Lischer & Excoffier 2012). For each individual, heterozygous sites were represented by one randomly selected allele.

*Population structure and relatedness analysis using RAD-seq data*

The VCF (pink_pigeon_subset.vcf) was used to generate sequences for each sample using SNPs called against the 100 longest contigs. The base from the reference assembly was used at positions where there is no SNP. If there was a homozygous SNP, then this alternative nucleotide was used. If the position was heterozygous, then the base was chosen at random. BEAST (Bouckaert et al. 2014) XML files were generated from the multiple-FASTA file for each contig using BEASTGen (<http://beast.community/beastgen>). BEAST (v2.5.1) (Bouckaert et al. 2014) was used to produce NEXUS tree files using a Yule tree prior and a strict molecular clock. The GTR + G substitution model was selected based on the AIC result from jModelTest v2.1.7 (Darriba et al. 2012). The Markov Chain Monte Carlo (MCMC) was run for 1x10^6^ generations with a sample frequency of 2,000. A single maximum clade credibility tree was produced in NEXUS format from each of the NEXUS files above using TreeAnnotator (v1.7.5). The NEXUS files were concatenated to produce a large NEXUS file containing a tree for each contig. The trees were visualised using DensiTree (Bouckaert 2010). Tracer v1.6 (<http://tree.bio.ed.ac.uk/software/tracer/>) was used to assess the effective sample size (ESS) of all estimated parameters (>200), as well as mixing and convergence of the MCMC to stationarity.

We investigated patterns of recent coancestry between sequenced pink pigeon samples using fineRADstructure (Malinsky et al. 2018) with default settings and the complete set of 43,967 SNPs. SNPs provided to fineRADstructure were ordered based on their genomic coordinates. In addition, VCFtools v0.1.15 (Danecek et al. 2011) was used to calculate average *F_ST_* across all 43,967 SNPs for all possible pairwise comparisons of geographically defined populations.

*Vortex simulations*

We simulated the impact of reintroducing captive birds into the free-living subpopulations using the population viability analysis software VORTEX 10.1 (Lacy & Pollak 2014). Vortex is an individual-based stochastic modelling software package to evaluate the impact of demographic, environmental and genetic factors on extinction risk (Kim et al. 2016; Lacy et al. 2020). Three different management scenarios were tested. The model was parameterised based on the empirically derived genetic load of lethal equivalents (LE = 15.13; see Fig. 4B), the estimated inbreeding coefficient, and the life history data (Appendix S3a). Briefly, Scenario 1 represents the free-living population without supplementation. In Scenario 2 (demographic rescue), the free-living population was supplemented from a hypothetical captive population which had the same alleles and allele frequencies as the free-living population. Scenario 3 simulates the impact of supplementing the free-living populations with individuals from the captive zoo populations which possess novel alleles. Reintroduction of these birds would reduce the mean kinship coefficient in the free-living population. Each scenario was run for a period of 100 years and model outputs were averaged across 1000 iterations.

Each scenario contained six populations, five of which represented the free-living subpopulations (Pigeon Wood (PW), Plaine Lievre (PL), Bel Ombre (BO), Ile aux Aigrettes (IAA), and Combo (CO)). The final population in each scenario was excluded from metapopulation calculations and represented either a captive population or a hypothetical population. The supplementation regime used was identical for both scenarios that included supplementation (Scenarios 2 and 3). Ten birds (five of each sex) were supplemented to each free-living subpopulation every five years using a staggered supplementation regime whereby each year a different subpopulation is supplemented.

By default, Vortex assumes that all populations start with an inbreeding coefficient *F* = 0 (i.e., no individuals are inbred). All pink pigeons (both free-living and captive) descend from a single population of around 20 individuals and display signs of inbreeding depression. Hence, we believe that this assumption is incorrect, and it would lead to an underestimation of the severity of inbreeding depression. Using the studbook data an average inbreeding coefficient was calculated for all captive birds in this study which had studbook numbers available (n= 44) using the software PMX 3.0.7 (Ballou et al. 2011), the mean inbreeding coefficient was *F* = 0.09 (Appendix S3d). Unlike the captive population, the free-living metapopulation has not been managed to reduce relatedness when breeding as such we predicted that the inbreeding coefficient of free-living birds would be higher and used the value of *F* = 0.15 (Swinnerton et al. 2004).

**Table S3a.** Parameters used in the Vortex simulations.

| Input Variable | Variable | | | | | | | | | | | | | | | | | | | Description | Reference |
| --- | --- | --- | --- | --- | --- | --- | --- | --- | --- | --- | --- | --- | --- | --- | --- | --- | --- | --- | --- | --- | --- |
| Scenario settings | | | | | | | | | | | | | | | | | | | | | |
| Number of iterations | 1000 | | | | | | | | | | | | | | | | | | | - | - |
| Number of years | 100 | | | | | | | | | | | | | | | | | | | - | - |
| Duration year in days | 365 | | | | | | | | | | | | | | | | | | | - | - |
| Extinction definition | one sex remains | | | | | | | | | | | | | | | | | | | - | - |
| Number of populations | 6* | | | | | | | | | | | | | | | | | | | - | - |
| Order of events | EV, Breed, Mortality, Disperse, Harvest, Supplement, Breed, Age | | | | | | | | | | | | | | | | | | | Pink pigeons can and do breed before a year old. To model this, another Breed event is included. This also provides the newly supplemented birds the opportunity to breed in the same year they are introduced. | Jones 1987 |
| Species Description | | | | | | | | | | | | | | | | | | | | | |
| *Lethal Equivalents* | 15.13 | | | | | | | | | | | | | | | | | | | These were calculated using logistic regression method with the small sample size correction. | Calculated from studbook data |
| *Percentage inbreeding due to LE* | 50% | | | | | | | | | | | | | | | | | | | Default | Lacy et al. 2020; Simmons & Crow 1977 |
| *Environmental Correlation between reproduction and survival* | 0.75 | | | | | | | | | | | | | | | | | | | Pink pigeons breed during most of the year and do not disperse far, and therefore, a good year for reproduction is also likely to be a good year for survival (barring catastrophes) | Jones 1987 |
| *Environmental correlation among populations* | 0.75 | | | | | | | | | | | | | | | | | | | All the free-living subpopulations are relatively geographically close (except Ile Aux Aigrette) and will be subject to similar environmental fluctuations | Jones 1987 |
|  |  | | | | | | | | | | | | | | | | | | |  |  |
| Dispersal  Concannon 2014 | | | | | | | | | | | | | | | | | | | | | |
| \|  \| **PW** \| **BF** \| **BO** \| **IAA** \| **CO** \| **Captive** \| \| --- \| --- \| --- \| --- \| --- \| --- \| --- \| \| **PW** \| 75.43 \| 4.17 \| 2.22 \| 0.18 \| 18.18 \| 0.00 \| \| **BF** \| 8.70 \| 87.66 \| 0.00 \| 0.00 \| 3.64 \| 0.00 \| \| **BO** \| 4.35 \| 0.00 \| 90.20 \| 0.00 \| 5.45 \| 0.00 \| \| **IAA** \| 0.00 \| 0.00 \| 2.22 \| 97.78 \| 0.00 \| 0.00 \| \| **CO** \| 26.09 \| 3.33 \| 11.11 \| 0.00 \| 59.47 \| 0.00 \| \| **Captive** \| 0.00 \| 0.00 \| 0.00 \| 0.00 \| 0.00 \| 100 \| | | | | | | | | | | | | | | | | | | | | | |
| Reproductive system | | | | | | | | | | | | | | | | | | | | | |
| *System* | Long term monogamy | | | | | | | | | | | | | | | | | | |  | Jones 1987 |
| *Age of first offspring female* | 1 | | | | | | | | | | | | | | | | | | | - | Jones 1987 |
| *Age of first offspring male* | 1 | | | | | | | | | | | | | | | | | | | - | Jones 1987 |
| *Max lifespan* | 15 | | | | | | | | | | | | | | | | | | | - | Jones 1987 |
| *Max age female reproduction* | 5 | | | | | | | | | | | | | | | | | | | - | Jones 1987 |
| *Max age male reproduction* | 15 | | | | | | | | | | | | | | | | | | | - | Jones 1987 |
| *Max number broods per year* | 12 | | | | | | | | | | | | | | | | | | | Pink pigeons have the ability to produce multiple broods per year particularly if a clutch fails. However, the number of successful broods is far lower than what they are capable of and there is a negative relationship between the number of broods and the number of fertile eggs an individual lays | Lind 1989 |
| *Max progeny per brood* | 2 | | | | | | | | | | | | | | | | | | | - | Jones 1987 |
| *Sex ratio at birth* | 54 | | | | | | | | | | | | | | | | | | | - |  |
| Reproductive rates | | | | | | | | | | | | | | | | | | | | | |
| *% adult female breeding* | 80 | | | | | | | | | | | | | | | | | | |  |  |
| *SD in % breeding due to EV* | 10 | | | | | | | | | | | | | | | | | | |  |  |
| *Distribution of broods per year* | 0  1  2  3  4  5  6  7 | | | | | Wild  9.52  30  50.96  7.14  2.38 | | | | | | | | Captive  36  20  10  10  5  5  14 | | | | | |  | Pink pigeon studbook; Concannon 2014 |
| *Distribution of number of offspring per brood* | 1  2 | | | | | 88  12 | | | | | | | | 78  22 | | | | | | The distribution of number of offspring per wild brood was taken from the original PHVA conducted in 1991. Whereas the figures for the captive population were calculated from studbook data. | Seal & Bruford 1991; pink pigeon studbook |
| Mortality | | | | | | | | | | | | | | | | | | | | | |
|  | PW | | BF | | | | BO | | | | IAA | | | | CO | | | | CA |  |  |
| *Mortality 0-1* | 54.4 | | 54.4 | | | | 54.4 | | | | 54.4 | | | | 54.4 | | | | 34 | The total mortality of individuals includes mortality due to inbreeding depression† This figure represents the mortality of moderately inbred birds from hatching to fledging. | Swinnerton et al. 2004 |
| *SD in mortality 0-1 due to EV* | 5 | | 5 | | | | 5 | | | | 5 | | | | 5 | | | | 5 | - | Seal & Bruford 1991 |
| *Mortality 1+* | 14.45 | 11.65 | | | | 19.4 | | | | 12.8 | | | | 15.6 | | | | 15 | | - | Bunbury 2006 |
| *SD in mortality 1+ due to EV* | 7.25 | 6.27 | | | | 5.55 | | | | 9.17 | | | | 4.80 | | | | 5 | | - | Bunbury 2006 |
| Initial population size | | | | | | | | | | | | | | | | | | | | | |
| *PW* | 70 | | | | | | | | | | | | | | | | | | | 2010 estimates, which are the most recent comprehensive estimates available | Concannon 2014 |
| *PL* | 115 | | | | | | | | | | | | | | | | | | |  |  |
| *BO* | 50 | | | | | | | | | | | | | | | | | | |  |  |
| *IAA* | 60 | | | | | | | | | | | | | | | | | | |  |  |
| *CO* | 50 | | | | | | | | | | | | | | | | | | |  |  |
| *Captive* | 79 | | | | | | | | | | | | | | | | | | |  |  |
| *Age distribution* | Stable | | | | | | | | | | | | | | | | | | |  |  |
| Carrying capacity (K)  Jones 1987; Seal & Bruford 1991 | | | | | | | | | | | | | | | | | | | | | |
| *PW* | 200 | | | | | | | | | | | | | | | | | | | The carrying capacity of the free-living metapopulation is higher than would naturally occur because MWF provide supplementary food to the pink pigeons which increases the carrying capacity and 200 is thought to be ideal. Except for the Ile Aux Aigrette which is limited by it being a closed population with a limited number of territories | Seal & Bruford 1991; Jones 1987 |
| *PL* | 200 | | | | | | | | | | | | | | | | | | |  |  |
| *BO* | 200 | | | | | | | | | | | | | | | | | | |  |  |
| *IAA* | 80 | | | | | | | | | | | | | | | | | | |  |  |
| *CO* | 200 | | | | | | | | | | | | | | | | | | |  |  |
| *Captive* | 500 | | | | | | | | | | | | | | | | | | |  |  |
| Catastrophes | | | | | | | | | | | | | | | | | | | | | |
| *Cyclones* | 6.7% | | | | | | | | | | | | | | | | | | | Cited as a major cyclone once every 15 years | Jones 1987 |
| Harvest  Harvest and supplementation are closely linked in vortex. To supplement from the captive population, it is necessary to first harvest form it. There has been some mortality observed from supplementations which is accounted for in supplementation. The supplementation/ harvest routine decided upon was one considered achievable (given past reintroductions). | | | | | | | | | | | | | | | | | | | | | |
| *Percent survival during translocation* | 100 | | | | | | | | | | | | | | | | | | | - | - |
| *Population harvested* | CA | | | | | | | | | | | | | | | | | | | - | - |
| *First year of harvest* | 1 | | | | | | | | | | | | | | | | | | | - | - |
| *Last year of harvest* | 99 | | | | | | | | | | | | | | | | | | | - | - |
| *Interval between harvests* | 5 | | | | | | | | | | | | | | | | | | | - | - |
| *Number of each sex to be harvested from age 0-1* | 20 | | | | | | | | | | | | | | | | | | | - | - |
| *Number of each sex to be harvested after age 1* | 5 | | | | | | | | | | | | | | | | | | | - | - |
| Supplementation | | | | | | | | | | | | | | | | | | | | | |
| *Percent survival during translocation* | 89.87 | | | | | | | | | | | | | | | | | | | - | Concannon 2014 |
| *Population supplemented* | PW | | | BF | | | | BO | | | | IAA | | | | CO | | | | - |  |
| *First year of supplement* | 5 | | | 6 | | | | 7 | | | | 8 | | | | 9 | | | | - |  |
| *Last year of supplement* | 95 | | | 96 | | | | 97 | | | | 98 | | | | 99 | | | | - |  |
| *Number of each sex to be supplemented from age 0 - 1* | 4 | | | 4 | | | | 4 | | | | 4 | | | | 4 | | | | Juvenile birds (< 6 months) are preferred for translocations which is why there are a greater number of younger birds being supplemented. The total number of birds chosen to be supplemented was an arbitrary choice because supplementation regimes are not the primary focus of this paper. However, the proposed numbers for the model are based on historical reintroduction attempts, and this number of birds can be taken from the captive population without significantly depleting it. | Concannon 2014; Zuel per comms. 2017 |
| *Number of each sex to be supplemented after age 1* | 1 | | | 1 | | | | 1 | | | | 1 | | | | 1 | | | |  |  |
| Genetics | | | | | | | | | | | | | | | | | | | | | |
| *Number of neutral loci to be modelled* | 22 | | | | | | | | | | | | | | | | | | | - |  |
| *Loci to be included in summary statistics* | Additional loci only | | | | | | | | | | | | | | | | | | | Only include the statistics from our data otherwise Vortex will include analyses of the alleles it has modelled by default. |  |
| *Number of loci to be subject to mutation* | 22 | | | | | | | | | | | | | | | | | | | - |  |
| *Mutation rate* | 0.0001 | | | | | | | | | | | | | | | | | | | - | (Harrisson et al. 2016) |
| *Start populations with all inbreeding and kinships set to* | PW  0.15 | BF  0.15 | | | BO  0.15 | | | | IAA  0.15 | | | | CO  0.15 | | | | CA  0.10/0.15 | | | Calculated based on inbreeding coefficients generated from the studbook data by the software PMX§ | (Swinnerton et al. 2004), Studbook |

*The last population (in this case the captive population) is excluded from metapopulation calculations so that the metapopulation modelled represents the free-living metapopulation

†Vortex models inbreeding depression as a reduction in first year survival which is calculated by the following equation:

S = S_0-1_*(e^-b(1-Pr[Lethals])F^)

In this equation, S is the probability of survival, S_0-1_ is the probability of survival from age 0-1 (i.e. from hatching to fledging), b is the number of lethal equivalents per haploid genome , Pr[Lethals] is the proportion of inbreeding depression due to lethal alleles and F the inbreeding coefficient (Lacy & Traylor-Holzer 2017). If the probability of first year mortality is 0.544 then (1-0.544) = 0.456, the probability of surviving the first year. This gives an overall first year survival probability of: Prob(Survival) = 0.456 * (e ^–(8.18*0.5*0.15)^) = 0.753 or 75.3%

**Appendix S3b.** Pedigree of the birds in the Ile aux Aigrettes (IAA) population reconstructed using the RAD-seq data.

**Appendix S3c.** Pedigree of the birds in the Ile aux Aigrettes (IAA) population reconstructed using the studbook data.

**Appendix S3d.** Mean inbreeding coefficient (*F*) calculated based on the studbook data of the pink pigeon.

**Appendix S3e.** Results from Vortex simulations and VortexR (Lacy & Pollak 2014; Pacioni & Mayer 2017) analysis for three different management scenarios where the probability of extinction within 100 years is most likely without genetic rescue from captive birds. Scenario 1 has been used as the baseline scenario to compare the effect of the different management plans. All results are the average of the 1000 iterations.

| **Scenario (#)** | **Probability of extinction in 100 years** | **Mean time to extinction over 100 years** | **Mean abundance year 100** | | **Mean heterozygosity year 100** | |
| --- | --- | --- | --- | --- | --- | --- |
|  |  |  | **SMMD** | **P value** | **SMMD** | **P value** |
| 1. No supplementation | 0.9990 | 54.4 | - | - | - | - |
| 2. Supplement with free-living | 0.8270 | 82.9 | 0.2820 | 0.3890 | 0.5644 | 0.2862 |
| 3. Supplement with captive | 0.0250 | 95 | 1.6890 | 0.0456 | 6.8973 | 0.0000 |

**Appendix S3f.** Linear regression of observed heterozygosity (*H_o_*) against the day of hatching after 16/01/1994 (the date of the first hatched bird in the dataset) in the a) IAA, b) Pigeon Wood, c) Combo, d) Plaine Lievre, and e) the Bel Ombre population, respectively. Shown are the regression lines of populations that showed a statistically significant decline. For the IAA population (a), the 95% confidence interval of the regression line (in blue) and 95% predictor interval of all data points (in red) is also shown. The results of the regression analysis are displayed in Table S1.

**Appendix S3g.** The relationship between the pedigree-calculated inbreeding coefficient (X-axis) versus the observed heterozygosity of individuals (Y-axis) for 109 birds of the IAA population for which both RAD-seq data and paternity data were available. Although there is a strong negative relationship between the level of inbreeding assessed using pedigrees and the genome-wide heterozygosity (Regression: F_1,108_=45.02, p<0.0001, R^2^-adj=29.0%), the pedigree information captures less than 30% of variation in gene diversity between individuals.

**Appendix S3h.** Weir and Cockerham *F_ST_* estimates between pink pigeon populations.

| **Comparison** | ***F_ST_*** |
| --- | --- |
| CO x BO | 0.016 |
| IAA x BO | 0.043 |
| IAA x CO | 0.045 |
| IAA x PL | 0.054 |
| IAA x PW | 0.049 |
| PL x BO | 0.016 |
| PL x CO | 0.030 |
| PW x BO | 0.009 |
| PW x CO | 0.014 |
| PW x PL | 0.024 |
|  |  |

**
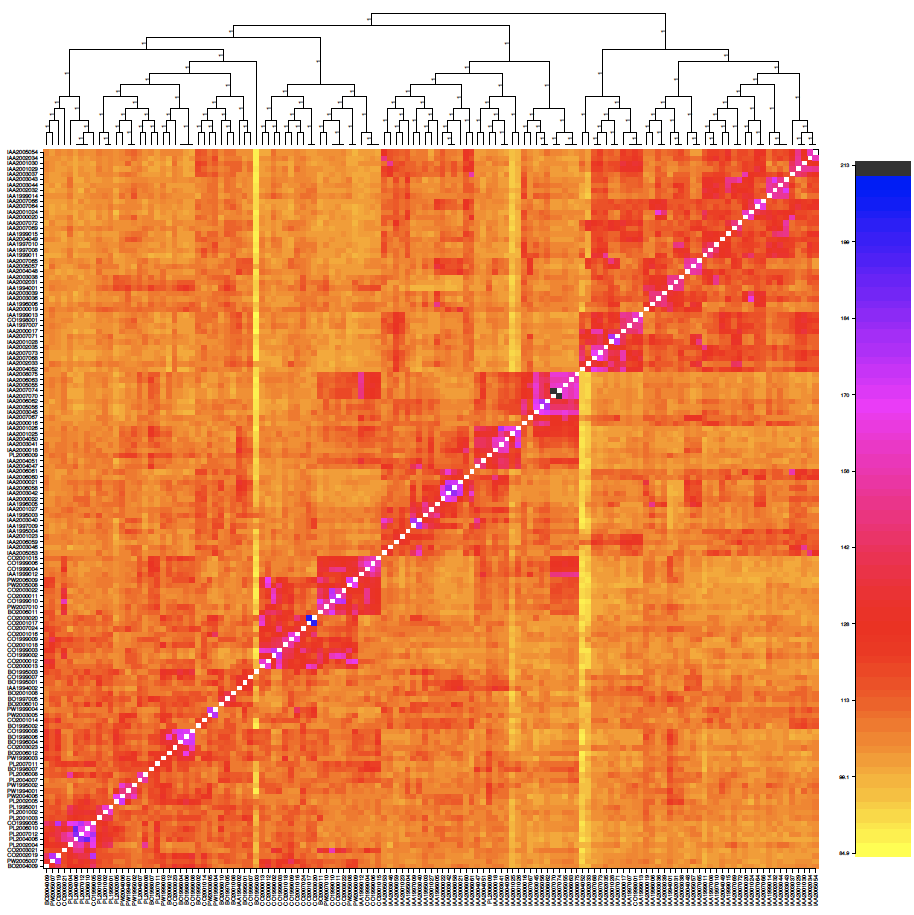
Appendix S3i.** Clustered fineRADstructure (Malinsky et al. 2018) co-ancestry matrix for all samples based on the full set of 43,967 SNPs. Geographically defined populations show weak clustering and overall there is a lack of well-defined clusters based on patterns of co-ancestry.

**Appendix S3j.** DensiTree (Bouckaert 2010) showing shared topologies based on sequences generated for each (post-2006) sample displayed. The figure is based on SNPs in the 100 largest contigs (bp) in the pink pigeon genome assembly. BEAST v2.5.1 (Bouckaert et al. 2014) was used to produce a phylogenetic tree for each contig using a multi-FASTA file including all samples. The figure shows the phylogenetic relationship between individuals for different contigs (grey lines). Topologies that are well supported by a relatively large set of subtrees are visible as darker outlines, and these represent topologies of individuals that are closely related. For example, 2007-IAA-070, 2007-IAA-074 and 2007-IAA-075 are three full siblings, and 2007-IAA-064 and 2007-IAA-066 are also full siblings, according to the IAA studbook. More distant family relationships can also be discerned; for example, 2007-IAA-058 is the uncle of 2007-IAA-061. The figure also identifies more complicated, multigenerational relationships, for example between 2007-IAA-074 (s337/6A00447) and 2007-IAA-068 (s274/6A00442). Based on pedigree information, the mother of the ancestor of 2007-IAA-074 is related to 2007-IAA-068 in the sixth generation (Male 507, Female 190), as well as in the third generation (Male 498, Female 520). At the father’s side, 2007-IAA-074 (s337/6A00447) is related to 2007-IAA-068 (s274/6A00442) in the seventh generation (Male is 958, Female is 307), as well as in the fourth and fifth generation (Male is 498, Female is 520) (there are two generations here, because the grandfather and the great grandmother are full siblings). The various topologies also illustrate variation in the level of similarity across different regions of the genome. Furthermore, the figure illustrates that such shared topologies appear even for apparently unrelated individuals from different populations. This demonstrates that inbreeding is inevitable in the post-bottleneck free-living pink pigeon population. It also explains why individuals from the same population do not form distinct clades in the network analysis (see Fig. 2F).


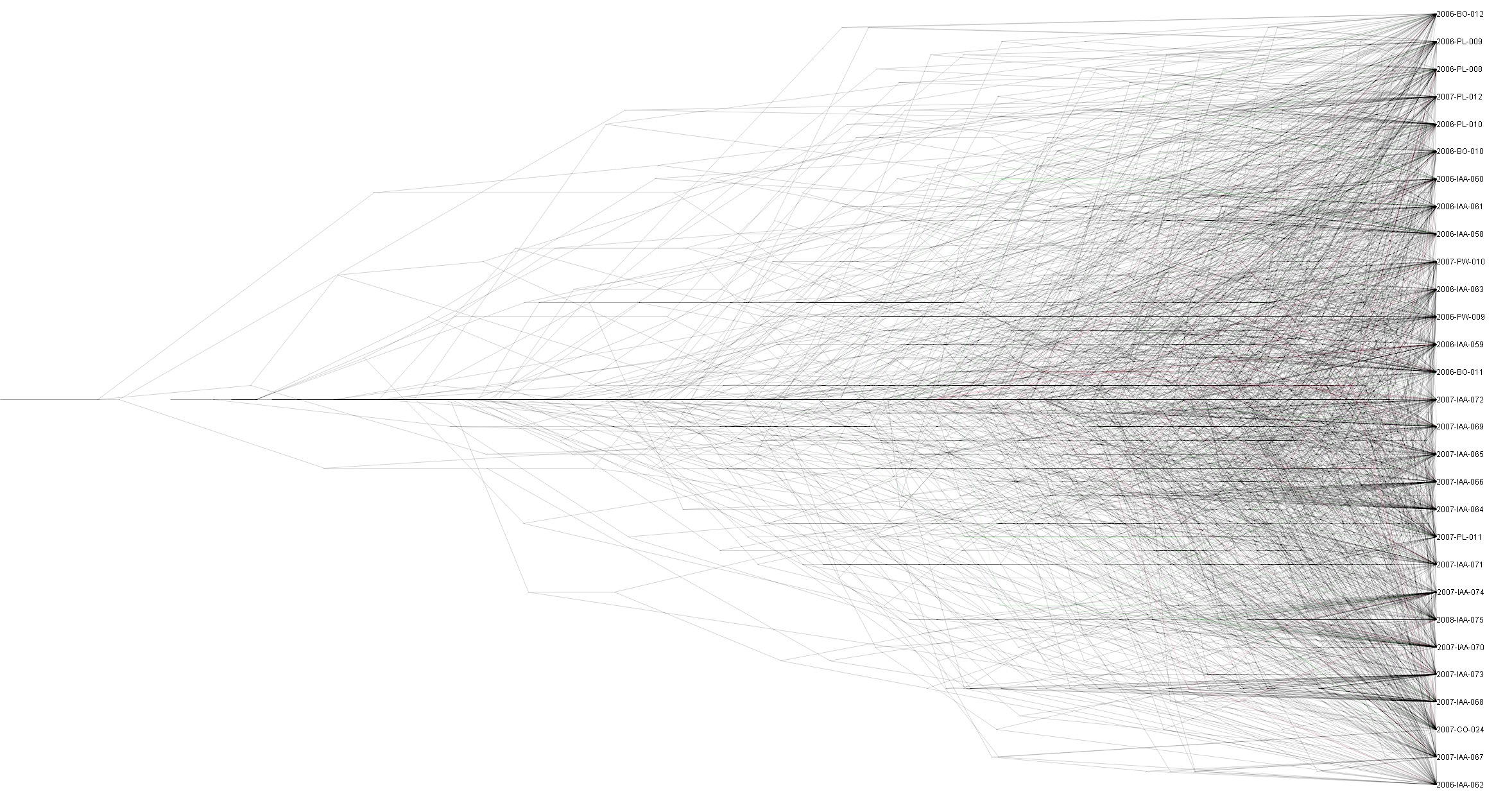


**Appendix S3k.** The criteria used to evaluate extinction risks in Aves, this shows that criteria E (the only criteria which allows for quantitative analyses) has never been used in the threat assessment of a bird species in the IUCN Red List. The data used here is from all threatened birds (Vulnerable, Endangered, Critically Endangered, n = 2534) that have been assessed by the IUCN Red List at the time of accessing the data on the 23^rd^ of August 2021 from <https://www.iucnredlist.org/>.

**Appendix S3l: Range of values used in sensitivity analysis and their reasons for inclusion.**

The "Base" scenario copies its values from Scenario 1 (no supplementation).

*Adult mortality varies for each subpopulation however during sensitivity testing the mean mortality for all five subpopulations (excluding Captive) was used as a baseline scenario as the aim was to examine the impact of altering adult mortality on the metapopulation but not individual subpopulations}.

** pink pigeons can have up to 2 offspring per brood, this parameter alters the percentage of females having a single offspring as opposed to two offspring, therefore 50 represents half the females having two offspring, and the other half of the female pink pigeons having a single offspring.

| Parameter | Base | Range | Interval | Reason for inclusion |
| --- | --- | --- | --- | --- |
| Adult mortality* | 14.78 | 10 - 30 | 5 | The data used is over 10 years old (Bunbury, 2006) therefore it is possible that they are not representative of the wild population as it is today. Because the primary causes of death varies between juveniles and adults (Bunbury *et al.*, 2007; Concannon, 2014), knowledge about the impact of mortality rates independent of life-stage may indicate where conservation should be focused (Pacioni and Mayer, 2017) |
| Juvenile mortality | 54.40 | 30 - 60 | 10 | These data were generated from a single subpopulation (IAA) several years ago because this is the only subpopulation that these data can be feasibly collected (Concannon, 2014). As such there is a chance that they may not accurately represent the other subpopulations or current mortality rates in IAA. |
| % of birds that have 1 offspring** | 88 | 50 - 100 | 10 | Inbreeding depression has been linked to reduced fertility in pink pigeons (Swinnerton *et al.*, 2004), if this parameter proves sensitive it would add support for the need for genetic rescue. |
| Carrying capacity | 200 | 50 - 250 | 50 | It is difficult to estimate carrying capacity that may also have been elevated artificially by supplementary feeding (Jones, 1987). If it is a significant factor in the pink pigeon's survival further research would be needed to ensure reserves are large enough to maintain viable populations |
| Number of LE | 15.13 | 3 -21 | 3 | LE are often not calculated and instead the default for Vortex is used (Harrisson *et al.*, 2016) however several studies have found their models sensitive to the number of LE modelled (King, Chamberlan and Courage, 2014; Licht, Moen and Romanski, 2017). The number of LE were calculated using longevity data but the number of LE can be estimated independently for a range of life history traits and then the results summed (O’Grady *et al*., 2006) By focusing solely on longevity the number of LE in pink pigeons may have been underestimated. |

**Appendix 3m.** Results from single factor sensitivity analysis. Scenarios which produced a significant difference in the abundance (Nall) of the pink pigeon after 100 years compared to the baseline scenario (no supplementation) are shown in bold.

| Parameter | Baseline value | Sensitivity analysis value | SSMD* | *P* |
| --- | --- | --- | --- | --- |
| Adult Mortality | 14.78 | 10 | 0.4472 | 0.3274 |
| Adult Mortality | 14.78 | 15 | -0.0607 | 0.4758 |
| Adult Mortality | 14.78 | 20 | -0.0637 | 0.4746 |
| Adult Mortality | 14.78 | 25 | -0.0637 | 0.4746 |
| Adult Mortality | 14.78 | 30 | -0.0637 | 0.4746 |
| Juvenile mortality | 54.40% | **30%** | **7.9449** | **0.0000** |
| Juvenile mortality | 54.40% | **40%** | **3.4692** | **0.0003** |
| Juvenile mortality | 54.40% | 50% | 0.511 | 0.3047 |
| Juvenile mortality | 54.40% | **60%** | -0.0637 | 0.4746 |
| % of birds that have 1 offspring | 88% | **50%** | **2.5769** | **0.0050** |
| % of birds that have 1 offspring | 88% | 60% | 1.2502 | 0.1056 |
| % of birds that have 1 offspring | 88% | 70% | 0.5749 | 0.2827 |
| % of birds that have 1 offspring | 88% | 80% | 0.1712 | 0.4321 |
| % of birds that have 1 offspring | 88% | 90% | -0.054 | 0.4785 |
| % of birds that have 1 offspring | 88% | 100% | -0.0637 | 0.4746 |
| Carrying capacity | 200 | 50 | -0.0637 | 0.4746 |
| Carrying capacity | 200 | 100 | -0.0637 | 0.4746 |
| Carrying capacity | 200 | 150 | -0.0622 | 0.4752 |
| Carrying capacity | 200 | 250 | -0.0193 | 0.4923 |
| Number of LE | 15.13 | **3** | **9.1258** | **0.0000** |
| Number of LE | 15.13 | **6** | **6.5468** | **0.0000** |
| Number of LE | 15.13 | **9** | **2.8943** | **0.0019** |
| Number of LE | 15.13 | 12 | 0.575 | 0.2827 |
| Number of LE | 15.13 | 15 | -0.0559 | 0.4777 |
| Number of LE | 15.13 | 18 | -0.0637 | 0.4746 |
| Number of LE | 15.13 | 21 | -0.0637 | 0.4746 |

*Strictly standardised mean difference is a measure of effect size, and it is calculated using the equation:

$SSMD_{i}=\frac{V_{i}- V_{B}}{\surd(s_{i}^{2}+ s_{B}^{2})}$,

where V_i_ and V_B_ are the mean value of the variable of interest in the *i*-th and the baseline scenarios respectively and s is the standard deviations of the parameter being measured (Zhang 2007). The sign of the SSMD indicates whether the scenario produces an increase in the probability of extinction (-ve) or a decrease in the probability of extinction (+ve). All the significant scenarios resulted in a decrease in the probability of extinction compared to the baseline scenario.

**Appendix literature cited**

Andrews, S. (2010). FastQC: A quality control tool for high throughput sequence data. Babraham Institute, Cambridge. Available from https://www.bioinformatics.babraham.ac.uk/projects/fastqc/ (accessed December 2013).

Aronesty, E. (2011). ea-utils: Command-line tools for processing biological sequencing data. University of Florida, Gainesville. Available from https://github.com/ExpressionAnalysis/ea-utils (accessed December 2013).

Baird, N. A., Etter, P. D., Atwood, T. S., Currey, M. C., Shiver, A. L., Lewis, Z. A., Selker, E. U., Cresko, W. A., & Johnson, E. A. (2008). Rapid SNP discovery and genetic mapping using sequenced RAD markers. *PloS one*, *3*(10), e3376. DOI:10.1371/journal.pone.0003376

Ballou, J. D., Lacy, R. C., Pollak, J. P. (2011). PMx: Software for Demographic and Genetic Analysis and Management of Pedigreed Populations (Version 1.0). Chicago Zoological Society, Brookfield. Available from http://www.vortex9.org/PMx.html (accessed January 2019).

Bouckaert R. R. (2010). DensiTree: making sense of sets of phylogenetic trees. *Bioinformatics, 26*(10), 1372–1373. DOI:10.1093/bioinformatics/btq110

Bouckaert, R., Heled, J., Kühnert, D., Vaughan, T., Wu, C. H., Xie, D., Suchard, M. A., Rambaut, A., & Drummond, A. J. (2014). BEAST 2: a software platform for Bayesian evolutionary analysis. *PLoS Computational Biology*, *10*(4), e1003537. DOI:10.1371/journal.pcbi.1003537

Bruford, M. W., Krupa, A. P., Burke, T. (1991). Genetic variation in the Jersey captive population of the pink pigeon (*Nesoenas mayeri*) revealed by DNA ﬁngerprinting. Pink pigeon conservation viability assessment. Minnesota: IUCN/SSC Captive-breeding Specialist Group

Bunbury, N. (2006). *Parasitic disease in the endangered Mauritian pink pigeon*. Doctoral Thesis. University of East Anglia.

Bunbury, N., Barton, E., Jones, C. G., Greenwood, A. G., Tyler, K. M. & Bell, D. J. (2007) Avian blood parasites in an endangered columbid: *Leucocytozoon marchouxi* in the Mauritian Pink Pigeon *Columba mayeri*. *Parasitology*, 134(6), 797-804. DOI:10.1017/S0031182006002149.

Concannon, L. (2014). *Managing threatened species: understanding the factors limiting the recovery of the endangered pink pigeon*. Doctoral Thesis. University of Reading.

Danecek, P., Auton, A., Abecasis, G., Albers, C. A., Banks, E., DePristo, M. A., Handsaker, R. E., Lunter, G., Marth, G. T., Sherry, S. T., McVean, G., Durbin, R., & 1000 Genomes Project Analysis Group (2011). The variant call format and VCFtools. *Bioinformatics*, *27*(15), 2156–2158.

Darriba, D., Taboada, G. L., Doallo, R., & Posada, D. (2012). jModelTest 2: more models, new heuristics and parallel computing. *Nature Methods*, *9*(8), 772. DOI:10.1038/nmeth.2109

Harrisson, K. A., Pavlova, A., Gonçalves da Silva, A., Rose, R., Bull, J. K., Lancaster, M. L., Murray, N., Quin, B., Menkhorst, P., Magrath, M. J. L., & Sunnucks, P. (2016). Scope for genetic rescue of an endangered subspecies though re-establishing natural gene flow with another subspecies. *Molecular Ecology*, 25(6), 1242-1258. DOI:10.1111/mec.13547.

Jones, C. G. (1987). The larger land-birds of Mauritius, in Diamond, A. W. (ed.) Studies of Mascarene Island Birds. Cambridge: Cambridge University Press, pp. 208–300.

Kim, B. J., Lee, B. K., Lee, H., & Jang, G. S. (2016). Considering threats to population viability of the endangered Korean long-tailed goral (*Naemorhedus caudatus*) using VORTEX. *Animal Cells and Systems* DOI:10.1080/19768354.2015.1127856.

King, T., Chamberlan, C., & Courage, A. (2014). Assessing reintroduction success in long-lived primates through population viability analysis: western lowland gorillas *Gorilla gorilla gorilla* in Central Africa. *Oryx*, 48(02), 294-303. DOI:10.1017/S0030605312001391.

Lacy, R. C., & Pollak, J. P. (2014). Vortex: A stochastic simulation of the extinction process. Version 10.0. Chicago Zoological Society, Brookfield. Available from http://www.vortex9.org/PMx.html (accessed January 2019).

Lacy, R. C., Miller, P.S., & Traylor-Holzer, K. (2020). Vortex 10 User’s Manual. IUCN SSC Conservation Planning Specialist Group, and Chicago Zoological Society, Apple Valley, Minnesota, USA.

Leggett, R. M., Ramirez-Gonzalez, R. H., Clavijo, B. J., Waite, D., & Davey, R. P. (2013). Sequencing quality assessment tools to enable data-driven informatics for high throughput genomics. *Frontiers in Genetics*, *4*, 288. DOI:10.3389/fgene.2013.00288

Li, H., Handsaker, B., Wysoker, A., Fennell, T., Ruan, J., Homer, N., Marth, G., Abecasis, G., Durbin, R., & 1000 Genome Project Data Processing Subgroup (2009). The Sequence Alignment/Map format and SAMtools. *Bioinformatics*, *25*(16), 2078–2079. DOI:10.1093/bioinformatics/btp352

Li H. (2011). A statistical framework for SNP calling, mutation discovery, association mapping and population genetical parameter estimation from sequencing data. *Bioinformatics*, *27*(21), 2987–2993. DOI:10.1093/bioinformatics/btr509

Li, H. (2013). Aligning sequence reads, clone sequences and assembly contigs with BWA-MEM. *arXiv preprint* arXiv:1303.3997v2.

Licht, D. S., Moen, R. A., & Romanski, M. (2017) Modeling viability of a potential canada lynx reintroduction to Isle Royale National Park. *Natural Areas Journal*, 37(2), 170–177. DOI:10.3375/043.037.0206

Lind, C. (1989). The effects of multiple clutching on the size, fertility and hatchability of the eggs of the pink pigeon (*Nesoenas mayeri*) at the Jersey Wildlife Preservation Trust. *Dodo*, 26, 93-98.

Lischer, H. E., & Excoffier, L. (2012). PGDSpider: an automated data conversion tool for connecting population genetics and genomics programs. *Bioinformatics*, *28*(2), 298–299.

Malinsky, M., Trucchi, E., Lawson, D. J., & Falush, D. (2018). RADpainter and fineRADstructure: Population inference from RADseq data. *Molecular Biology and Evolution*, *35*(5), 1284-1290. DOI:10.1093/molbev/msy023

O’Grady, J. J., Brook, B. W., Reed, D. H., Ballou, J. D., Tonkyn, D. W., & Frankham, R. (2006). Realistic levels of inbreeding depression strongly affect extinction risk in wild populations. *Biological Conservation* 133(1), 42–51. DOI:10.1016/j.biocon.2006.05.016.

Pacioni, C., & Mayer, R. vortexR: an R package for post Vortex simulation analysis. *Methods in Ecology and Evolution.* DOI:10.1111/2041-210X.12786.

Purcell, S., Neale, B., Todd-Brown, K., Thomas, L., Ferreira, M. A., Bender, D., Maller, J., Sklar, P., de Bakker, P. I., Daly, M. J., & Sham, P. C. (2007). PLINK: a tool set for whole-genome association and population-based linkage analyses. *American Journal of Human Genetics*, *81*(3), 559–575. DOI:10.1086/519795

Seal, U. S., Bruford, M. (1991). *Columba (Nesoenas) mayeri* pink pigeon conservation viability assessment workshop. Jersey Wildlife Preservation Trust: Captive breeding specialist group. Conservation Planning Specialist Group (CPSG), Apple Valley. Available from http://www.cbsg.org/sites/cbsg.org/files/documents/PinkPigeonPHVA_FInalReport.pdf.

Simmons, M. J., & Crow, J. F. (1977). Mutations affecting fitness in Drosophila populations. *Annual Review of Genetics*, *11*, 49–78. DOI:10.1146/annurev.ge.11.120177.000405

Swinnerton, K. J., Groombridge, J. J., Jones, C. G., Burn, R. W., & Mungroo, Y. (2004). Inbreeding depression and founder diversity among captive and free-living populations of the endangered pink pigeon *Columba mayeri*. *Animal Conservation* DOI:10.1017/S1367943004001556.

Zhang, X. D. (2007). A pair of new statistical parameters for quality control in RNA interference high-throughput screening assays. *Genomics,* 89(4), 552-561. DOI:10.1016/j.ygeno.2006.12.014.
