# Supplementary material for: Genomic erosion in a demographically recovered bird species during conservation rescue
Source: Conserv Biol. 2022 May 12;36(4):e13918. doi: 10.1111/cobi.13918 (PMC9546124; doi:10.1111/cobi.13918)
Supplement: Supplementary file 4 — Appendix A4 [file COBI-36-0-s004.docx]

**Appendix S4: SLiM simulations**

*SLiM simulations*

We performed individual-based forward simulations with SLiM v3.1 (Haller & Messer, 2019) to examine the impact of different management regimes on a slowly collapsing population that experienced a severe bottlenecked (Appendix S4a). Our simulations are based on a non-Wright-Fisher implementation, which considers overlapping generations, age-structure, and customizable offspring generation and migration patterns. During the simulation each time step consists of three stages: reproduction, dispersal (between captive and wild populations, if any), and mortality. Absolute fitness (i.e., probability of survival) was regulated by the carrying capacity and the known aged-based probability of mortality for pink pigeons (Appendix Table S3a and Figure S4a).

Genetics parameters: we simulated 4000 genes of 3,400 bp each distributed proportionally to the number of genes contained across 28 autosomal chromosomes in the collared flycatcher genome (Kawakami et al., 2014). We thus simulated a total genomic length of 13.6 Mb. We used a recombination rate of 1x10-4 per base-position per generation, with no recombination within genes. We simulated neutral and deleterious mutations at relative proportion of 1:2.3 with selection coefficients taken from a gamma distribution with shape of 0.5 and scale of 0.1, plus 5% of lethal mutations (Kardos et al., 2021). This distribution is consistent with the predicted fitness effects of deleterious variation in humans (Eyre-Walker & Keightley, 2007). Dominance coefficients (h) are poorly characterized in natural systems, but metanalysis indicates that deleterious mutations tend to be recessive, or partially recessive (Charlesworth & Willis, 2009) and experimental approaches reveal an inverse relationship between selection and dominance coefficients where highly deleterious mutations are mostly recessive (Agrawal & Whitlock, 2012). Thus, we followed the strategy implemented in Kyriazis, Wayne, and Lohmueller (2021) and Henn et al. (2016) to capture this inverse relationship (Appendix S4c) with the formula:

$$h=\frac{0.5}{1+7071.07*s}$$

To model the amount of deleterious variation observed in pink pigeons (~15 lethal equivalents; see Results) we used a mutation rate of 7.5e-8 in an ancestral population of 16,000 individuals (Ryan, 2021).

Demographic parameters: we simulated a demographic trajectory that capture the trend observed in the pink pigeon by controlling an overall carrying capacity informed by the inferred (pre-1980s) population size and recorded census trajectories since 1980 (Appendix S4a). We modelled a single panmictic wild population to reflect the observed low levels of inter-population differentiation. We also modeled a single captive population founded by 12 individuals in 1976, growing at the rate reported in GDEWS records until reaching an average of 120 individuals.

Life-history parameters: we controlled mating and mortality of individuals during the simulation. Every simulation step monogamic pairs of age 1+ individuals were formed at random, and their clutch size was drawn from a distribution that reflects the productivity of pink pigeons in the wild (see Appendix Table S3a). Mortality probability of individuals was determined according to their age class as reported in field data (Appendix Table S3a). Captive individuals were slightly more productive and had a lower probability of mortality. These parameters lead to an average generation time of 3.5 simulation steps, which is very close to the average generation time of four year in the pink pigeon, thus in our simulation each simulation step can be considered as one year.

Management scenarios: we tested three different scenarios after the population reached the bottom of the bottleneck (Appendix S4a); (1) a no intervention scenario where the wild population stays at the historical low size, (2) a demographic rescue scenario where the population grows to 400 individuals as recorded in the wild population without any contribution from the captive population, (3) a genetic rescue scenario where the wild population received translocations from the captive population, and (4) a demographic + genetic rescue scenario where the population grows to 400 individuals and receives translocations from the captive population. Translocations occurred at rates reported by GDEWS until year 2019 (light grey bars in Appendix S4a), thereafter at random to capture the same dynamics (dark grey bars in Appendix S4a).

Simulations: We performed the simulations in three stages. First, we ran a burn-in for 50,000 steps to obtain an ancestral population in mutation-selection-drift equilibrium. To speed up the burn-in period, we recorded the entire genealogy using Tree Sequence recording without mutations, and recapitated the trees to archive full coalescence into a single root and overlaid neutral mutations with pyslim and msprime (Haller, Galloway, Kelleher, Messer, & Ralph, 2019). We used the resulting tree as a starting point for the rest of the simulation adding the deleterious mutation which reach mutation-selection-drift equilibrium faster. We ran this second step with deleterious mutations for 20,000 steps. Finally, we ran these in-silico population in equilibrium through the declining demography trajectory. We performed 40 replicate runs for each scenario. In the main text we present results from year 1920 onwards, results for the full run (year 1773 onwards) are presented in the Appendices below.


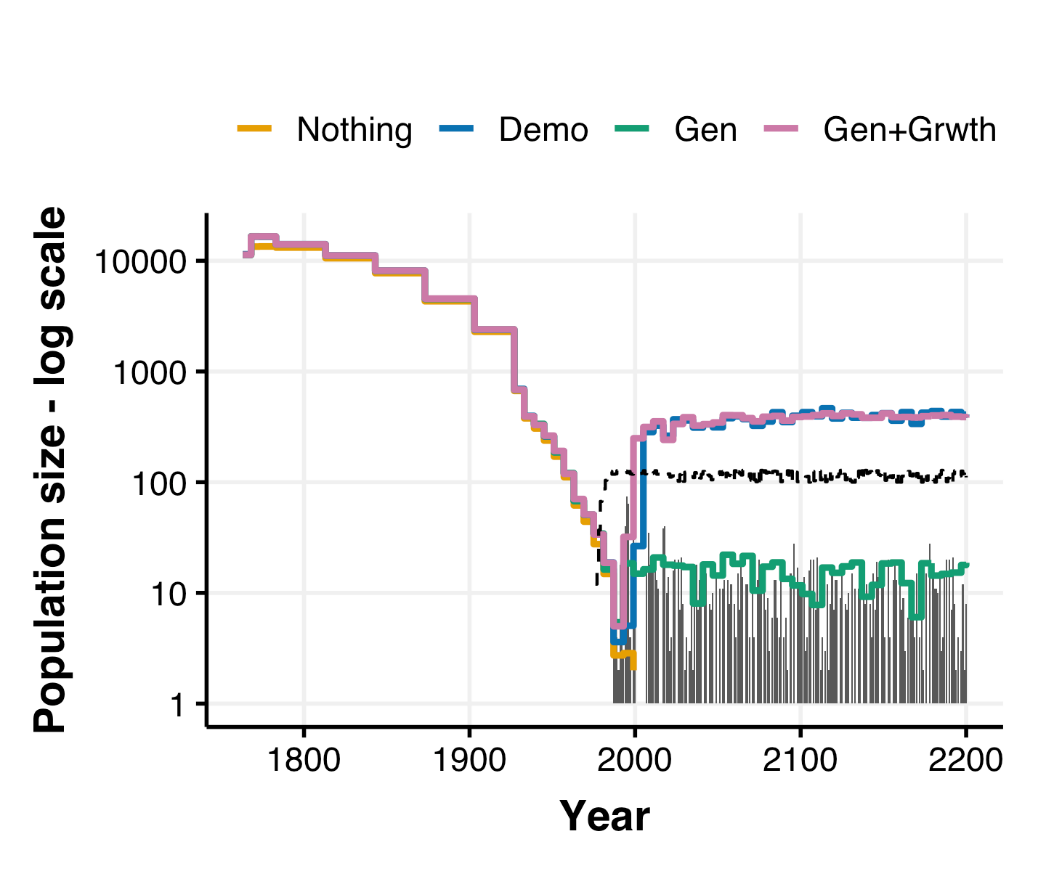


**Appendix S4a.** Demographic trajectory used in SLiM (Haller & Messer, 2019) simulations. Alternative management scenarios after bottleneck recovery are show in coloured lines: (1) yellow: no intervention, (2) blue: demographic rescue, (3) green: genetic rescue and (4) pink: demographic + genetic rescue. Translocation rates of captive individuals into the wild populations are shown in light grey bars for known records and dark grey bars for random numbers that capture the same dynamics thereafter. The dark dashed line represents the captive population of the GDEWS.

**Appendix S4b.** Life-history parameters used in the SLiM (Haller & Messer, 2019) simulations (a) Probability of mortality per age class (b) Probability of clutch-size per monogamic pair.

**Appendix S4c.** Negative relationship between dominance and selection coefficient based on yeast data from Agrawal and Whitlock (2012) Kyriazis et al. (2021) and Henn et al. (2016)


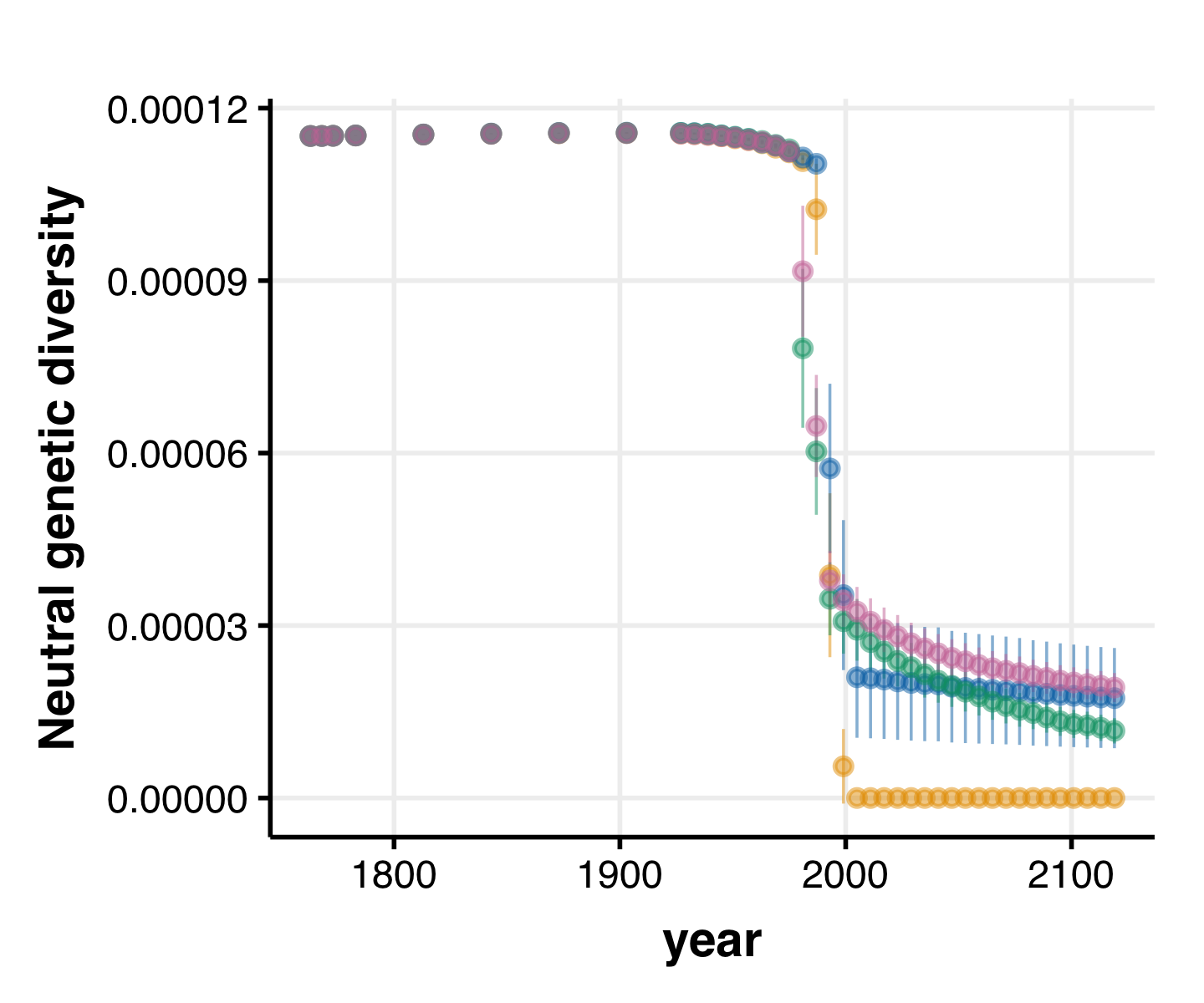


**Appendix S4d.** Mean (dot) and standard deviation (bar) of nucleotide diversity across 40 SLiM simulation replicates for four management scenarios (1) yellow: no intervention, (2) blue: demographic rescue, (3) green: genetic rescue and (4) pink: demographic + genetic rescue.


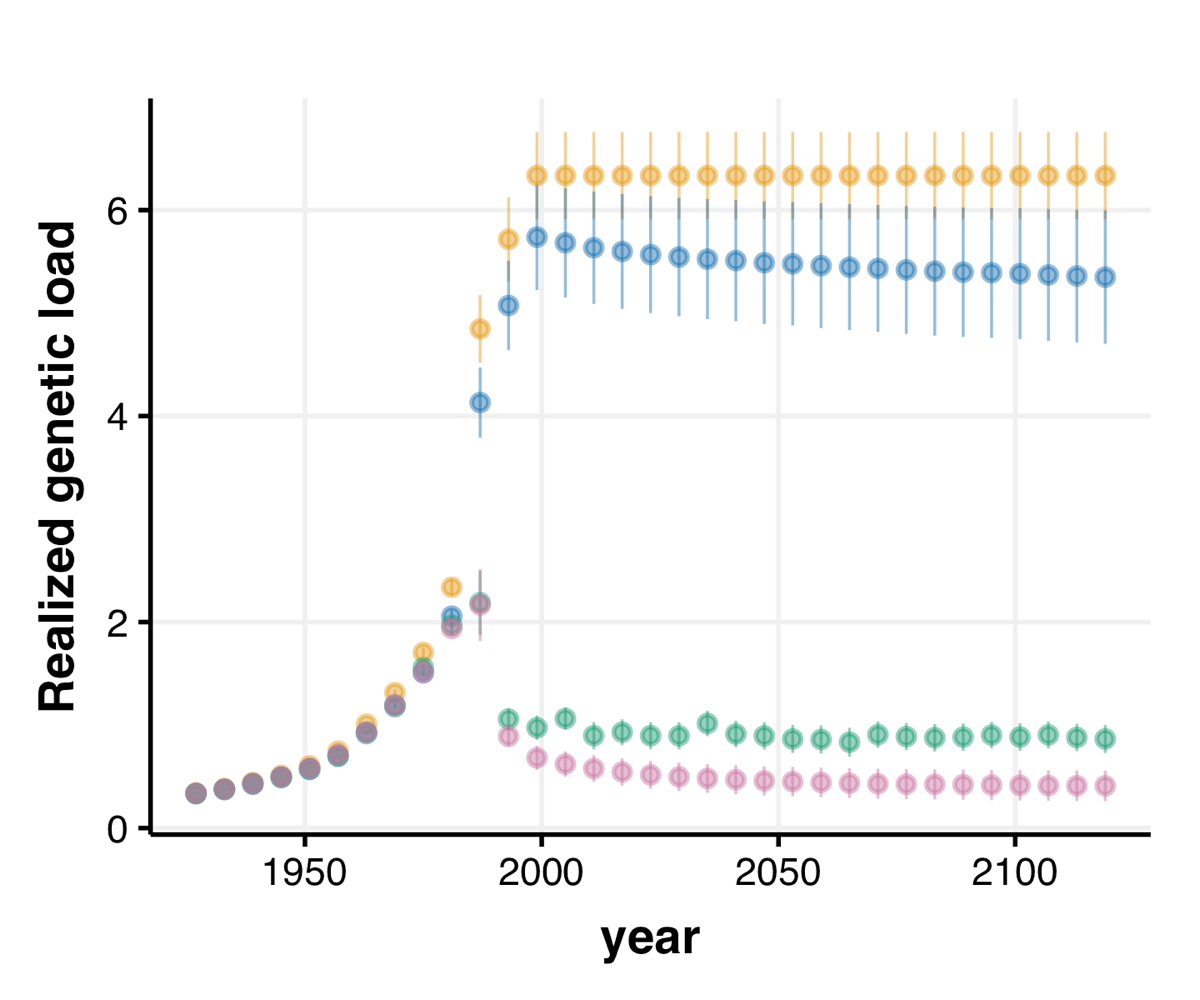


**Appendix S4e.** Mean (dot) and standard deviation (bars) of the fitness effect of the genetic load (i.e., realized load; Bertorelle et al.) across 40 SLiM simulation replicates for four management scenarios (1) yellow: no intervention, (2) blue: demographic rescue, (3) green: genetic rescue and (4) pink: demographic + genetic rescue.


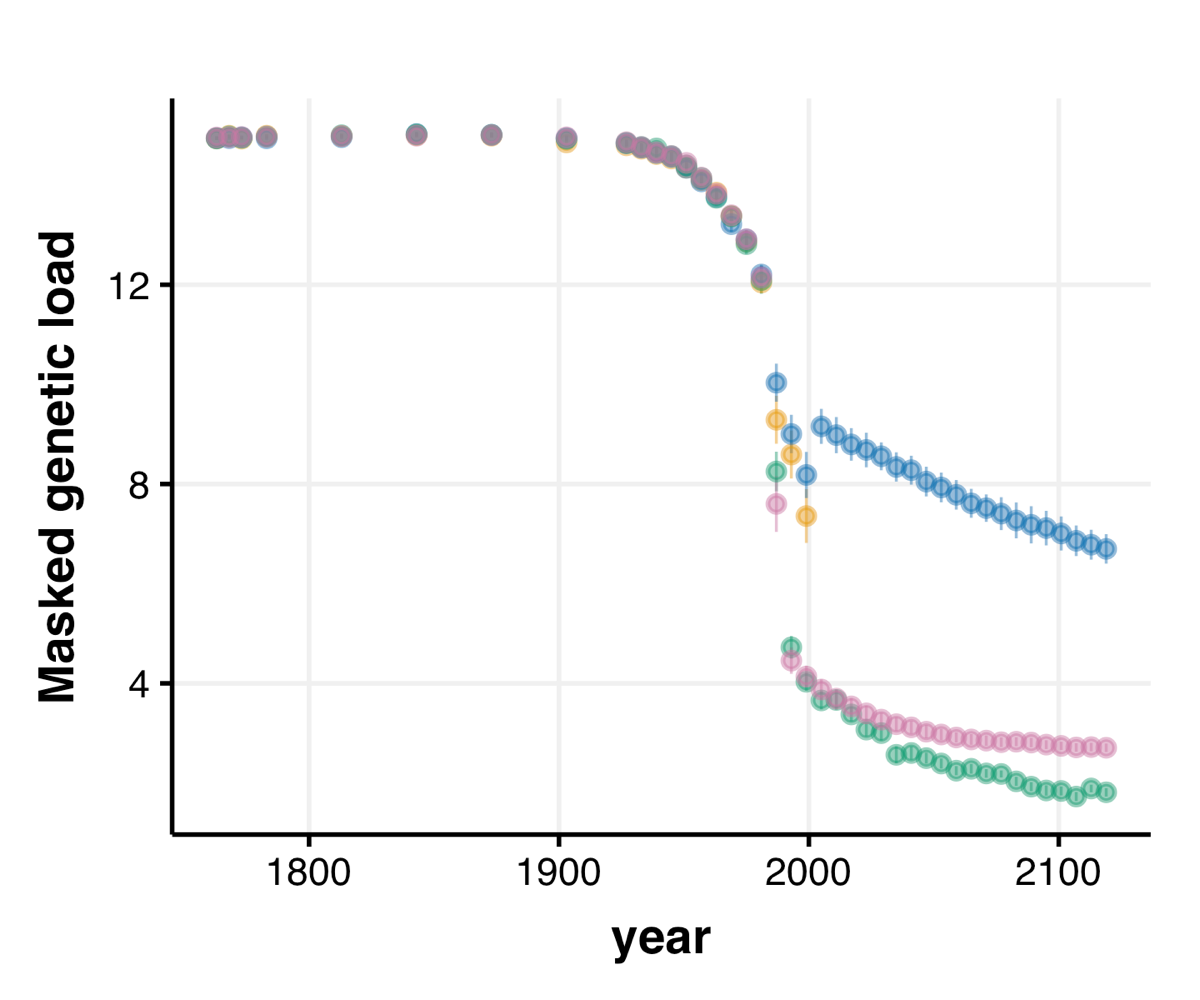


**Appendix S4e.** Mean (dot) and standard deviation (bars) for the component of the load that it is expressed on fitness effect (i.e., masked load; Bertorelle et al.) across 40 SLiM simulation replicates for four management scenarios (1) yellow: no intervention, (2) blue: demographic rescue, (3) green: genetic rescue and (4) pink: demographic + genetic rescue.

**Appendix literature cited**

Agrawal, A. F., & Whitlock, M. C. (2012). Mutation load: the fitness of individuals in populations where deleterious alleles are abundant. *Annual Review of Ecology, Evolution, and Systematics, 43*, 115-135.

Charlesworth, D., & Willis, J. H. (2009). The genetics of inbreeding depression. *Nature Reviews Genetics, 10*(11), 783-796.

Eyre-Walker, A., & Keightley, P. D. (2007). The distribution of fitness effects of new mutations. *Nature Reviews Genetics, 8*(8), 610-618.

Haller, B. C., Galloway, J., Kelleher, J., Messer, P. W., & Ralph, P. L. (2019). Tree‐sequence recording in SLiM opens new horizons for forward‐time simulation of whole genomes. *Molecular ecology resources, 19*(2), 552-566.

Haller, B. C., & Messer, P. W. (2019). SLiM 3: forward genetic simulations beyond the Wright–Fisher model. *Molecular Biology and Evolution, 36*(3), 632-637.

Henn, B. M., Botigué, L. R., Peischl, S., Dupanloup, I., Lipatov, M., Maples, B. K., . . . Snyder, M. P. (2016). Distance from sub-Saharan Africa predicts mutational load in diverse human genomes. *Proceedings of the National Academy of Sciences, 113*(4), E440-E449.

Kardos, M., Armstrong, E. E., Fitzpatrick, S. W., Hauser, S., Hedrick, P. W., Miller, J. M., . . . Funk, W. C. (2021). The crucial role of genome-wide genetic variation in conservation. *Proceedings of the National Academy of Sciences, 118*(48).

Kawakami, T., Backström, N., Burri, R., Husby, A., Olason, P., Rice, A. M., . . . Ellegren, H. (2014). Estimation of linkage disequilibrium and interspecific gene flow in *Ficedula flycatchers* by a newly developed 50k single‐nucleotide polymorphism array. *Molecular ecology resources, 14*(6), 1248-1260.

Kyriazis, C. C., Wayne, R. K., & Lohmueller, K. E. (2021). Strongly deleterious mutations are a primary determinant of extinction risk due to inbreeding depression. *Evolution Letters, 5*(1), 33-47.

Ryan, C. (2021). *Better tools, better resources, better conservation: integrating genome data into the conservation of the pink pigeon Nesoenas mayeri.* University of East Anglia,
